# Nonconsensual Sexual Experience Acknowledgment: Exploring the Roles of Gender Identity, Sexual Aggression Myths, and Psychological Inflexibility

**DOI:** 10.3390/bs15070875

**Published:** 2025-06-27

**Authors:** Wesley Malvini, Jessica M. Criddle, Mark S. Lacour, Emily K. Sandoz

**Affiliations:** 1 Department of Psychology, University of Louisiana at Lafayette, Lafayette, LA 70503, USA; wesley.maxwell-malvini1@louisiana.edu (W.M.); mark.lacour@louisiana.edu (M.S.L.); emily.sandoz@louisiana.edu (E.K.S.); 2 Louisiana Contextual Science Research Group, Lafayette, LA 70503, USA

**Keywords:** nonconsensual sexual experiences, sexual violence acknowledgment, psychological inflexibility, psychological flexibility, rape myths, gender identity

## Abstract

Nonconsensual sexual experiences (NSEs) can take many forms, including rape and sexual assault. NSE acknowledgment has been linked to several positive and negative outcomes. A person’s acknowledgment of their NSEs may be in part due to the extent to which they accept myths about sexual aggression (AMASA). However, AMASA does not fully account for NSE acknowledgment, which necessitates research on possible moderators. Further, other individual differences, such as gender identity, may play a role in both AMASA and acknowledgment. The primary aim of this study was to examine psychological flexibility (PF) and inflexibility (PI) as potential moderators of the relationship between NSE acknowledgment and AMASA. AMASA and gender identity both had significant, positive associations with acknowledgment. Furthermore, there was a significant negative relationship between acknowledgment and PI. The current study provides data on two psychological constructs that may serve as target areas for developing interventions to support people with NSE histories, as well as furthering our understanding of acknowledgment across gender identities.

## 1. Introduction

In the U.S., an incident of sexual violence occurs every 68 seconds (RAINN, n.d.). Sexual violence, or nonconsensual sexual experiences (NSEs; e.g., Kilimnik & Meston, 2020), takes many forms (e.g., rape, sexual assault) and occur across the gender spectrum. Over 25% of cisgender women and nearly 15% of cisgender men report being raped, and over 50% of women and 30% of men report other forms of NSEs throughout their lifetime (Basile et al., 2022). However, these data underrepresent the scope of the problem, as most incidents of sexual violence go unreported (Basile et al., 2022; World Population Review, 2023). One factor contributing to the low rates of acknowledgment in the literature may be that participants who do not report their NSEs in legal settings do not acknowledge them in research settings (Cleere & Lynn, 2013; Sasson & Paul, 2014). Thus, identifying variables influencing NSE acknowledgment and how they relate to clinical processes may be beneficial across settings.

### 1.1. Nonconsensual Sexual Experience Acknowledgment

Accurate reporting of sexual violence in the literature may be limited for various reasons. For example, people who have NSE histories may be uncertain about how to label their experiences or are reluctant to do so (Sasson & Paul, 2014). About 60.4% of people with NSEs do not use common sexual violence labels (i.e., do not acknowledge; Wilson & Miller, 2016). Understandings of terms like “survivor,” “victim,” “rape,” or “sexual assault” often vary across individuals (Rousseau et al., 2020). Furthermore, many people with NSEs prefer not to self-label, and imposed labels can have harmful effects (McMullin & White, 2006; Williamson & Serna, 2018).

The literature identifies three categories of NSE acknowledgment (e.g., Lipinski et al., 2021). The first is acknowledged, where people use common sexual violence labels (Donde et al., 2018; Koss, 1985). The second is unacknowledged, where people reject such labels (e.g., Cleere & Lynn, 2013; Wilson et al., 2016). The third is ambivalent acknowledgment, reflecting uncertainty due to conflicting thoughts, feelings, or context (Lipinski et al., 2021). Ambivalent acknowledgment may more accurately describe over half of people described as unacknowledged (Lipinski et al., 2021). Inconsistent findings in prior studies (e.g., Littleton et al., 2007; Wilson & Miller, 2016) may stem from measures that overlook the nuanced ways individuals label their experiences. Including ambivalent acknowledgment may improve measurement accuracy by addressing data invariance, low statistical power, and the limitations of dichotomous responses.

### 1.2. Factors Contributing to Acknowledgment

Several factors contribute to NSE acknowledgment (see Wilson & Miller, 2016). These include perpetration tactics (Lipinski et al., 2021), assailant gender (Artime et al., 2014), and others (e.g., Kubany & Manke, 1995; Rousseau et al., 2020). As of yet, there is a dearth of research examining the effects of perpetration tactics, so the exact processes by which and the extent of the effects are unknown. Although there is little research on the effects of assailant gender, the literature indicates that both cisgender men and women assaulted by females are less likely to acknowledge (Artime et al., 2014). Time elapsed since the event also consistently contributes to acknowledgment (e.g., Littleton et al., 2007), as stages of denial and ambivalence may occur before acknowledgment (Rousseau et al., 2020). Rape myth acceptance (RMA) and gender identity may also contribute to NSE acknowledgment (Peterson & Muehlenhard, 2004; Watters & Yalch, 2023).

### 1.3. Gender Differences in Acknowledgment

Gender identity is significantly associated with acknowledgment (e.g., Watters & Yalch, 2023). However, conclusions drawn from this literature are limited in two ways. First, NSE acknowledgment in cisgender men is understudied (Peterson et al., 2019). Second, most acknowledgment studies do not differentiate gender beyond the binary^1^ (i.e., cisgender men vs. cisgender women; e.g., Artime et al., 2014; Blayney et al., 2021). Low representation of gender nonbinary and transgender persons in study samples contributes to a dearth of research on these populations (see Coulter et al., 2015, 2017). Because of this, we included broader, more inclusive gender information in our questionnaires.

With regard to the binary gender differences in the literature, cisgender men acknowledge NSEs less often than cisgender women (e.g., Watters & Yalch, 2023). Even so, most cisgender women and girls with NSEs do not acknowledge (Donde et al., 2018; Newins et al., 2021; Rousseau et al., 2020). Specifically, more than 75% of cisgender men (e.g., Artime et al., 2014) and between 50 and 75% of cisgender women with NSEs are unacknowledged (Orchowski et al., 2013; Wilson et al., 2016; Wilson & Scarpa, 2017). Comparatively, those who do not conform to the gender binary appear to acknowledge at much higher rates (Anderson et al., 2021c). One reason for the differences in acknowledgment across gender identities may be due to RMA.

### 1.4. Rape Myth Acceptance

Rape myths are defined as stereotyped beliefs about those who have perpetrated or been victimized by NSEs (Burt, 1980). RMA refers to the degree to which a person endorses such beliefs (e.g., Crall & Goodfriend, 2016). RMA helps foster a culture that denies or reduces the perceived effects of NSEs while blaming those victimized for their victimization and admonishing their assailant (Burt, 1980; Sleath & Bull, 2010). Myths about sexual aggression (MASA) include the key components of rape myths while addressing a broader array of sexual violence experiences, such as nonpenetrative NSEs (Gerger et al., 2007). The acceptance of MASA (AMASA) refers to the degree to which a person endorses MASA and, although less studied, may pose the same risks as RMA alone (e.g., March et al., 2021; Samji & Vasquez, 2019).

The literature has yielded mixed results regarding the relationship between NSEs and RMA. For example, some report that NSEs do not predict RMA (Crall & Goodfriend, 2016; Mason et al., 2004), some report a positive association (e.g., Baugher et al., 2010), and others report a negative association (e.g., Nyul et al., 2021; Vonderhaar & Carmody, 2015). This suggests there are other factors influencing the relationship between acknowledgment and AMASA that have yet to be sufficiently explored. Like acknowledgment, RMA appears to be associated with the extent to which a person with NSEs encounters adverse mental health outcomes (see Suarez & Gadalla, 2010). For example, people with NSEs who are low in RMA report higher depression and PTSD symptoms compared to those who are high in RMA (Valdespino-Hayden et al., 2022). A person’s RMA is also negatively associated with NSE acknowledgment (Heath et al., 2013; Peterson & Muehlenhard, 2004). One reason cisgender men tend to have unacknowledged NSEs may be, at least in part, the result of greater AMASA (Artime et al., 2014; Reed et al., 2019; Sleath & Bull, 2010). 

The differences in acknowledgment across gender identities may be linked to RMA. For example, there is substantive evidence that cisgender women who do not acknowledge their NSEs are more likely to endorse rape myths than those who acknowledge them (Reed et al., 2019; Wilson & Newins, 2019). However, AMASA alone does not fully predict the lower rates of NSE acknowledgment found in cisgender men compared to other genders (Reed et al., 2019). AMASA also does not fully predict why gender-minoritized people tend to have higher rates of NSE acknowledgment than cisgender men and women (Wilson & Newins, 2019). Thus, further research is necessary to identify additional factors contributing to these relationships.

### 1.5. Psychological Flexibility

One such factor may be psychological flexibility (PF), a person’s ability to remain in contact with painful psychological experiences without judgment, avoidance, or struggles to decrease them while engaging in contextually sensitive behaviors in accordance with one’s personal values (Cherry et al., 2021; Rolfs et al., 2016). PF is a transdiagnostic construct contributing to a wide range of adaptive behaviors (Bond et al., 2011; Hayes et al., 2006) and is regarded as a critical component for healthy psychological functioning (e.g., Gloster et al., 2011). For example, PF has been found to have a negative association with depression and PTSD symptomatology (e.g., Bordieri et al., 2014; Fonseca et al., 2020). Additionally, PF may serve as a buffer between early life trauma and developing depression and PTSD symptoms in adulthood (Richardson & Jost, 2019). Thus, it may be worthwhile to examine the role of PF in the relationship between NSE acknowledgment and AMASA as a potentially critical target area to increase psychological well-being in people who are unacknowledged or ambivalent.

### 1.6. Psychological Inflexibility

Psychological inflexibility (PI) can be characterized as a pattern of rigid, contextually insensitive behavior of struggling to control or change internal events, such as unpleasant thoughts and emotions, in ways that interfere with behavior in line with one’s chosen values (Hayes et al., 2006). PI is associated with the development and maintenance of PTSD (Cheng et al., 2021; Meyer et al., 2019), as well as various other psychopathologies (Giommi et al., 2023; Morris & Mansell, 2018). While PF is associated with individual health and well-being and PI is associated with psychological distress (e.g., Rolfs et al., 2016), they are not distal ends of the same spectrum (e.g., Morris & Mansell, 2018), but rather two separate, yet related, repertoires (e.g., Cherry et al., 2021). In fact, any person can differ across dimensions of PF and PI independently (e.g., showing high levels of mindfulness while simultaneously being high in experiential avoidance; e.g., Cherry et al., 2021).

One core component driving PI is experiential avoidance (EA), which is also associated with a multitude of negative psychological effects. Specifically, EA of one’s NSEs appears to intensify the negative effects of victim-blaming responses (i.e., a form of AMASA) to NSE disclosure (i.e., of which NSE acknowledgment is one type) on depressive symptoms (Bhuptani et al., 2019). Additionally, EA can intensify a person’s psychological distress (e.g., Bardeen & Fergus, 2016) and has been linked to a greater risk of denial, self-blame, and impaired social functioning (Criddle et al., 2022; Karekla & Panayiotou, 2011). Using EA to minimize one’s distress is not always maladaptive (e.g., Bonanno & Burton, 2013; Griffin, 2022). In fact, it may provide temporary relief from distress (e.g., Hayes et al., 2004; Tyndall et al., 2018), especially when used as a coping strategy following a traumatic experience (Tull et al., 2004).

For instance, a person with an NSE history may avoid stimuli that remind them of their experience, including NSEs as a topic of conversation (Burrows, 2013). In this way, a lack of NSE acknowledgment may be predicted by higher levels of EA. Given the interdependent nature of the components of PI, PI may also contribute to a lack of acknowledgment, lower levels of reporting, depression, and PTSD symptoms. In other words, as NSE acknowledgment is known to be time-dependent, it is possible that the temporary relief characterizing PI contributes to lower rates of reported psychological dysfunction in people with unacknowledged compared to acknowledged NSEs. However, as the literature consistently demonstrates, attempting to alter or change the frequency or form of unpleasant internal experiences can be costly as time passes, such that undesired symptoms temporarily decrease and then increase (see Hayes et al., n.d.). Thus, as the long-term negative effects of EA come to surpass the benefits, one may then be more motivated and able to acknowledge and process an NSE, leading to greater negative symptomatology. Considering all of this, PF and PI may be important constructs to consider when investigating NSE acknowledgment relative to AMASA.

### 1.7. Methodological Gaps

There are several gaps in the existing literature on NSE acknowledgment status and AMASA stemming from methodology. For instance, most studies assume women have NSEs and men perpetrate them (Rousseau et al., 2020), with only a few studies breaking this trend (e.g., Artime et al., 2014; Nyul et al., 2021). The exclusion of men from NSE research is problematic as a more inclusive approach may lead to a deeper understanding of NSE outcomes and treatment efficacy (Anderson et al., 2020, 2023). Additionally, there is a dearth of research using gender minority samples (cf. Anderson et al., 2021c); some studies even exclude these participants entirely due to small sample sizes (e.g., Valdespino-Hayden et al., 2022).

Further, acknowledgment has traditionally been measured using a dichotomous response format (i.e., acknowledged, unacknowledged; see meta-analysis, Wilson & Miller, 2016). However, recent research (Lipinski et al., 2021) has included measurement for an ambivalent acknowledgment status by utilizing Likert-type rather than binary measures due to evidence suggesting a scaled response format yields significantly more people who acknowledge perpetrating sexual violence (e.g., Anderson et al., 2023, 2021b). There are currently no studies using a scaled response format in victimization studies, as most researchers transform the data into ordinal measurement types prior to analyses.

### 1.8. The Current Study

The primary aim of the current study is to advance the literature by examining PF and PI as potential moderators of the relationship between NSE acknowledgment and AMASA. A secondary aim of this study is to address methodological gaps in the literature with respect to the treatment of gender relative to NSE history and treat acknowledgment as a continuous variable rather than a categorical one. The findings of this study may provide a deeper understanding of the mechanisms influencing the acknowledgment of NSEs occurring since age 14. Furthermore, the current study is poised to provide preliminary data on two psychological constructs that may serve as target areas for developing interventions to provide support to those who have NSE histories.

This study had several hypotheses and exploratory research questions. First, we predicted AMASA would be positively associated with participants’ degree of NSE acknowledgment. Second, we predicted PF would be positively associated with participants’ degree of NSE acknowledgment. Third, we predicted PI would be negatively associated with participants’ degree of NSE acknowledgment. Fourth, we predicted gender identity would be related to NSE acknowledgment. Specifically, cisgender men would have the lowest degree of NSE acknowledgment, followed by cisgender women, and people with a gender minority identity would have the highest degree of NSE acknowledgment. Finally, we examined whether PF or PI moderates the relationship between NSE acknowledgment and AMASA and whether gender identity moderates the relationship between NSE acknowledgment and AMASA.

## 2. Methods

The current study and its associated research questions, hypotheses, procedures, measures, and analytic plan were preregistered with the Open Science Framework (OSF) prior to beginning data collection. The registration and all Supplementary Materials can be viewed at this link: https://doi.org/10.17605/OSF.IO/36VZF.

### 2.1. Participants

Participants (*N* = 1177) were sampled from three populations—a university in the southern United States using an experimental management system (SONA) available to undergraduate psychology students (*n* = 313, 26.6%), a nationally representative community sample using the crowdsourcing platform Prolific (*n* = 732, 62.2%; www.prolific.com) and two online sources (i.e., Facebook and Instagram; *n* = 132, 11.2%). Eligible participants were required to be over the age of 18 years and currently residing in the United States. Participants were required to endorse at least one of the behavioral descriptions of NSEs to be included in the final analysis. Participants with no NSE histories (*n* = 180, 19.8%) were allowed to participate in the study; however, their data was excluded from the final analysis. For a detailed overview of participant flow based on recruitment methods, please see Figure S1 in the Supplementary Materials.

The final analysis involved a sample of 697 participants ranging from 18 to 72 years of age (*M* = 31.96, *SD* = 12.26). Our sample was predominantly White/European American (*n* = 461, 66.1%) and straight/heterosexual (*n* = 465, 66.7%) who endorsed either currently being enrolled in college or having completed some college or higher (*n* = 547, 78.5%). Furthermore, we identified 506 participants as cisgender women (72.6%), 132 as cisgender men (18.9%), 56 as having a gender minority identity (8.0%), and 3 who declined to identify (0.4%). For a complete breakdown of our sample characteristics, please see Table 1.

### 2.2. Procedures

Prior to data collection, all procedures were approved by the university’s Institutional Review Board (Protocol IRB-24-024-PSYC). Data collection occurred between April and September of 2024. Participants recruited using SONA received partial course credit as compensation. Online participants were not compensated for their time. Finally, participants recruited through Prolific received USD $8 per hour for their participation (*M* = USD $2.67). All participant responses were confidential and not linked to their identity at any point. Participants were credited by means of an alternative unique identifier generated by the respective participant management application.

Participants were given a link to access the survey hosted on Qualtrics (www.qualtrics.com). Those who agreed to the informed consent and met all eligibility criteria were administered a battery of randomly ordered self-report questionnaires. A demographics questionnaire was then administered. Following the demographic questionnaire, participants were thanked for their time and provided with study disclosures and resources for mental health treatment, sexual violence advocacy, sexual violence awareness, and other related resources.

### 2.3. Measures

Demographics Questionnaire. The demographic questionnaire was created in alignment with the American Psychological Association (APA)’s Equity, Diversity, and Inclusion (EDI) framework (Akbar & Parker, 2021). Participants provided their age, sex assigned at birth, gender identity, sexual identity, and race or ethnicity. The researchers provided options allowing each participant to “prefer not to respond” and to “prefer to self-describe.”

Post-Refusal Sexual Persistence Scale—Victimization (PRSPS-V; Struckman-Johnson et al., 2019). The revised version of the PRSPS-V, which uses gender-neutral language, was used to identify eligible participants by assessing NSEs histories from the age of 14 years (Anderson et al., 2021a). Participants were asked to indicate how many times they have experienced any of the included 24 behavioral descriptions of NSEs, with response options of 0, 1, 2–5, 6–9, and 10+. Participants who endorsed one or more items on the PRSPS-V were considered to have an NSE history. Reliability for PRSPS-V scored dichotomously was found to be acceptable (*κ* = 0.60–0.68) for both men and women (Anderson et al., 2021a). The PRSPS has also been found to have good construct validity (Struckman-Johnson et al., 2003). For the current study, the PRSPS had excellent internal consistency (*α* = 0.91). Most studies on acknowledgment are conducted only with participants aged 14 and over. This is potentially due to higher prevalence rates starting at age 14 (Finkelhor et al., 2009), age ranges used in common measurement development (Finkelhor et al., 2014), ethical considerations of asking pediatric populations about NSEs in a research setting (Walsh et al., 2004), and that some research shows a positive linear relationship between age and NSE disclosure (McElvaney et al., 2020).

Nonconsensual Sexual Experience Acknowledgment. Two additional 5-point Likert-scaled items were added to the PRSPS to assess participants’ NSE acknowledgment. The items asked participants whether they had ever been sexually assaulted (item 1) or raped (item 2) since the time they were 14 years old. The language in these items was taken directly from a previously established measure; the only modification was moving from a binary response option (yes/no) to a scaled format (0 = definitely not, 4 = definitely yes; Watters & Yalch, 2023). Higher scores on the acknowledgment item indicate greater confidence in NSE acknowledgment. The final acknowledgment variable was created using participants’ highest score from the two items; the final variable reflects their highest level of surety in applying a sexual violence label to their NSEs.

The Acceptance of Modern Myths about Sexual Aggression Scale, Revised (AMMSA-R; Malvini, 2025). The AMMSA-R asks participants to read 16 statements related to modern myths about sexual aggression and rate how much they agree with each of them by selecting an option from a 7-point Likert scale (1 = completely disagree, 7 = completely agree). We revised the AMMSA-21 (Bohner et al., 2022) to exclude the subscale lack of support for policies designed to help alleviate the effects of sexual violence (e.g., “Nowadays, the victims of sexual violence receive sufficient help in the form of women’s shelters, therapy offers, and support groups”) and added an item that states “a man can only be raped by another man.” Participant scores were determined by averaging scores across all items (Bohner et al., 2022), with higher scores indicating greater acceptance of sexual aggression myths. The AMMSA-21 has been found to have high internal consistency and criterion validity (*α* = 0.93; Bohner et al., 2022), and the AMMSA-R had excellent internal consistency in this sample (*α* = 0.92).

The Multidimensional Psychological Flexibility Inventory (MPFI; Rolfs et al., 2016). The MPFI assesses the six distinct factors making up PF and the six comprising PI. Participants were asked to indicate how true each of the 60 statements was for them over the last two weeks using a 5-point scale ranging from 1 = never true to 6 = always true. Global composite scores for either PF or PI were calculated by averaging the means for each of the six respective dimension subscales. The MPFI has shown high levels of internal consistency (MPFI-PF [*α* = 0.96–0.97]; MPFI-PI [*α* = 0.95–0.96]), as did this sample (MPFI-PF [*α* = 0.96]; MPFI-PI [*α* = 0.96]), and high levels of both convergent and discriminant validity (Rolfs et al., 2016).

### 2.4. Analytic Procedures

All statistical analyses were conducted using RStudio 2024.04.2 + 764 (R Core Team, 2021). Because it was not possible to leave items without responding to them, there were no incomplete responses.

Tests of normality were run. A Shapiro–Wilk test showed that the MPFI-PF (*W* = 0.99, *p* = 0.198) was normally distributed. However, the AMMSA-R (*W* = 0.98, *p* < 0.001), MPFI-PI (*W* = 0.99, *p* < 0.001), and NSE acknowledgment (*W* = 0.81, *p* < 0.001) had non-normal distributions; visual inspection showed negative skewness for all. All variables were standardized, with the exception of NSE acknowledgment. The distribution of our NSE acknowledgment data was comparable to other researchers (see Wilson & Miller, 2016); thus, to retain important information and for comparability with binary acknowledgment scales in the literature, the variable was not transformed. Further, multicollinearity was assessed using Variance Inflation Factor (VIF) and tolerance values; values for all variables fell within normal ranges with no VIFs greater than 4 or tolerances lower than 0.78.

To examine whether regression outliers affected any of our analyses, we retested all models, excluding observations with a Cook’s *D* value greater than 4/(*n* − *k* − 1), in line with recommendations from Fox (2008). In nearly all instances, the models excluding outliers using Cook’s *D* showed larger effect sizes. Thus, the analyses reported in our results reflect those with outliers removed. The results for these analyses with outliers included are reported in the tables provided in the Supplementary Materials. Further, we used an approximation of the Bayes Factor (BF; Wagenmakers, 2007) following initial analyses to assess whether non-significant findings required more data to adjudicate between the null and alternative hypotheses or whether the data at hand strongly favored the null compared to the alternative.

## 3. Results

### 3.1. Missing Acknowledgment Data

Due to an unforeseen technical error with Qualtrics, no data were collected to assess if participants used the label “rape” concerning acknowledgment status in the online sample. Thus, acknowledgment for these 49 participants could only be determined based on the sexual assault label. We conducted a chi-square between the acknowledgment item for sexual assault and rape for people with an NSE history who responded to both items to determine if there was a difference between either using the highest score between the sexual assault item and the rape item to create our acknowledgment variable or simply using the sexual assault item alone to determine participant’s degree of acknowledgment. The results were not statistically significant (*χ*^2^[4] = 4.20, *p* = 0.38), indicating no differences in the representation of either sexual violence label relative to acknowledgment. Thus, all 49 participants with missing responses to the rape label were retained for analysis.

### 3.2. Descriptive Statistics

We conducted a Pearson’s *r* correlation analysis between all normally distributed measures (Table 2) and found the MPFI-PF was positively correlated with the AMMSA-R (*r* = 0.21, *p* < 0.001) and participant age (*r* = 0.12, *p* < 0.001). The MPFI-PI was negatively correlated with the AMMSA-R (*r* = −0.07, *p* = 0.048), participant age (*r* = −0.27, *p* < 0.001), and the MPFI-PF (*r* = −0.44, *p* < 0.001). The AMMSA-R was negatively correlated with participant age (*r* = −0.11, *p* = 0.002). We conducted Kendall’s *tau* correlation analysis with NSE acknowledgment due to skewness. We found NSE acknowledgment had a significant positive correlation with the MPFI-PI (*τ* = 0.14, *p* < 0.001) and participant age (*τ* = 0.08, *p* = 0.003) and a significant negative correlation with the AMMSA-R (*τ* = −0.17, *p* < 0.001).

For a detailed breakdown of the proportion of acknowledging, ambivalently acknowledging, and unacknowledging participants in our sample, as well as the frequency of sexual violence labels (i.e., rape, sexual assault) selected, please see Table S1 in the Supplementary Materials.

### 3.3. Hypothesis Testing

First, we examined the associations between acknowledgment and the variables AMASA, PF, and PI using a series of simple linear regressions of each predictor variable on the outcome variable. We then conducted a one-way, between-subjects analysis of variance (ANOVA) on acknowledgment using gender identity as the predictor variable with three levels (cisgender men, cisgender women, and people with a gender minority identity), followed by pairwise *t*-tests using Bonferroni corrections.

**Figure 1:**
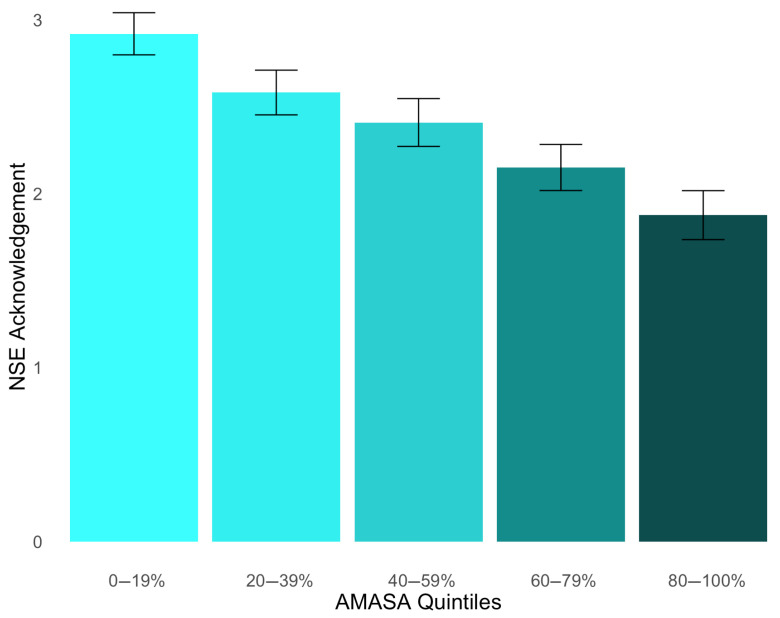
Degree of nonconsensual sexual experience (NSE) acknowledgment across quintiles of acceptance of myths about sexual aggression (AMASA). Each quintile (i.e., bin) illustrates 20% of the data distribution for the variable on the *x*-axis with the lowest range of scores in bin 1 (0–19%) and the highest range of scores in bin 5 (80–100%); error bars represent standard error of the mean (SEM); the mean AMASA scores were 2.92 for bin 1 (*SD* = 1.43), 2.58 for bin 2 (*SD* = 1.52), 2.41 for bin 3 (*SD* = 1.62), 2.15 for bin 4 (*SD* = 1.56), and 1.88 for bin 5 (*SD* = 1.65).

To test Hypothesis 1, that AMASA is positively associated with participants’ degree of NSE acknowledgment, we performed a linear regression (see Table S2 in Supplementary Materials). Final scores on the AMMSA-R were regressed onto the degree of NSE acknowledgment. Our hypothesis was supported, *b* = −0.52, *p* < 0.001, 95% CI [−0.64, −0.40] (see Figure 1). Since *BF*_10_ > 100, our results strongly favored the alternative over the null hypothesis.

**Figure 2:**
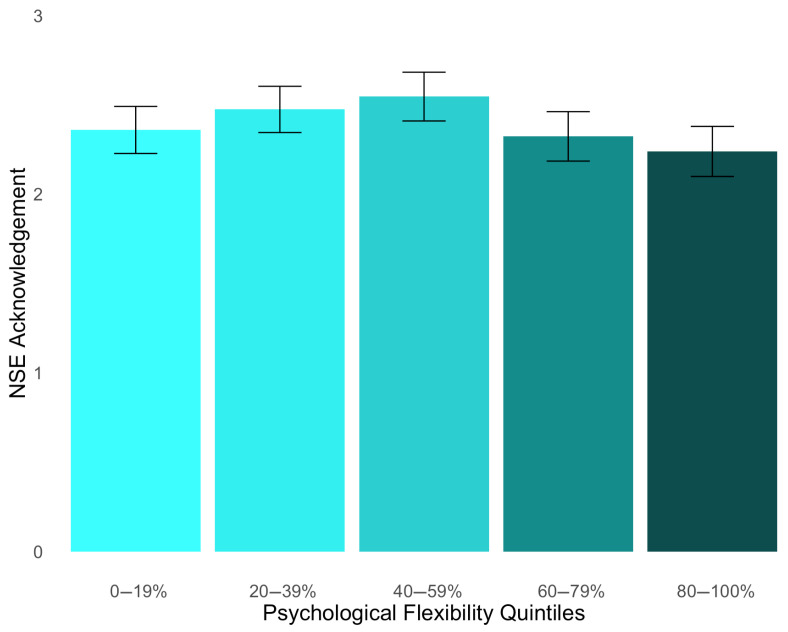
Degree of NSE acknowledgment across quintiles of psychological flexibility (PF). The mean PF scores were 2.36 for bin 1 (*SD* = 1.56), 2.47 for bin 2 (*SD* = 1.53), 2.55 for bin 3 (*SD* = 1.61), 2.32 for bin 4 (*SD* = 1.63), and 2.39 for bin 5 (*SD* = 1.65).

Hypothesis 2 predicted that PF would have a positive relationship with a degree of NSE acknowledgment. This hypothesis was tested with a linear regression (see Table S3 in Supplementary Materials), wherein our independent variable was global PF scores, and our dependent variable was a degree of NSE acknowledgment. Our hypothesis was not supported, *b* = −0.06, *p* = 0.373, 95% CI [−0.19, 0.07] (see Figure 2). The results of the BF estimation (*BF*_01_ = 17.43) verified that our data strongly favored the null hypothesis over the alternative.

**Figure 3:**
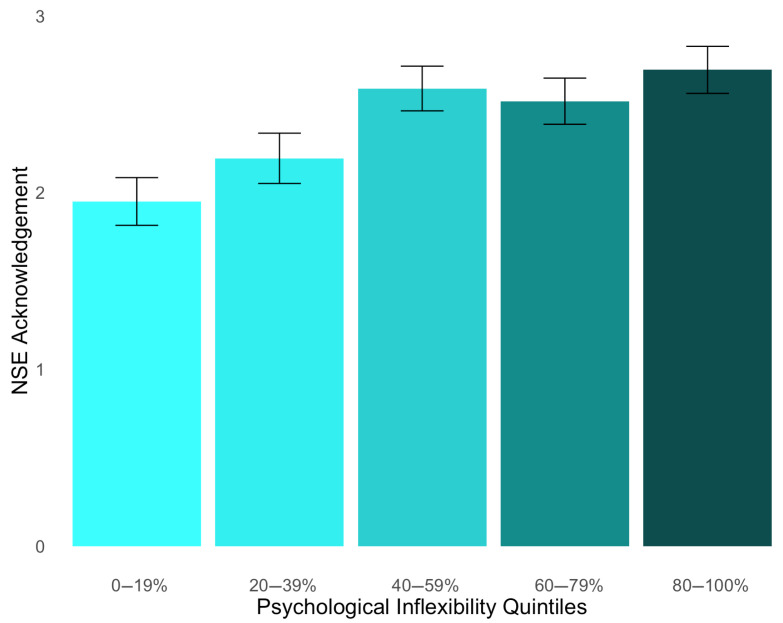
Degree of NSE acknowledgment across quintiles of psychological inflexibility (PI). The mean PI scores were 1.95 for bin 1 (*SD* = 1.59), 2.19 for bin 2 (*SD* = 1.68), 2.59 for bin 3 (*SD* = 1.49), 2.52 for bin 4 (*SD* = 1.54), and 2.70 for bin 5 (*SD* = 1.56).

A linear regression analysis was performed to test Hypothesis 3, which states that PI has a negative relationship with the degree of NSE acknowledgment (see Table S4 in Supplementary Materials). Our independent variable was global PI scores, and our dependent variable was a degree of NSE acknowledgment. While our analysis indicated a statistically significant relationship between PI and acknowledgment, it was in the opposite direction of what was expected, *b* = 0.41, *p* < 0.001, 95% CI [0.29, 0.53] (see Figure 3). Since *BF*_10_ > 100, our results strongly favored the alternative over the null hypothesis.

**Figure 4:**
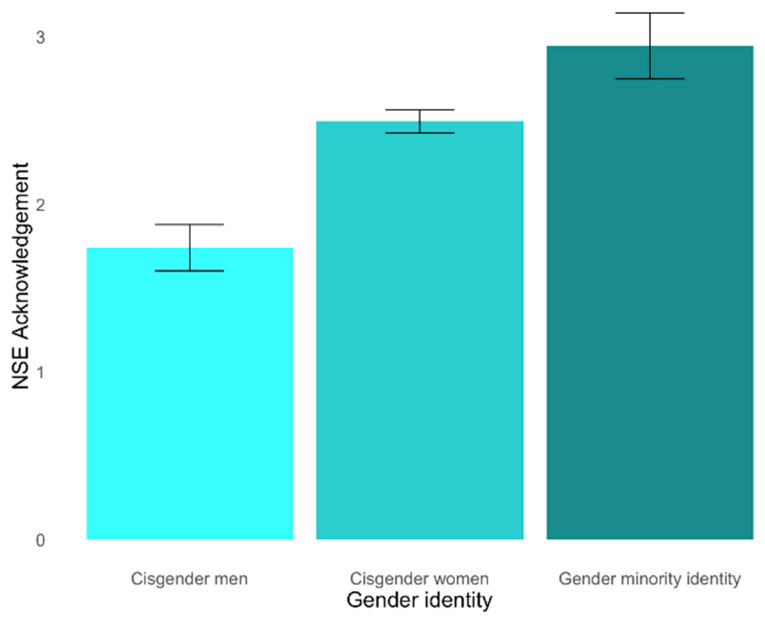
Differences in the degree of NSE acknowledgment across gender identities.

To test Hypothesis 4, which states that gender identity differentiates the degree of NSE acknowledgment, we first conducted a one-way, between-subjects ANOVA. Our dependent variable was a degree of NSE acknowledgment, and our independent variable was gender identity, which had three levels: cisgender men, cisgender women, and people with a gender minority identity. We removed observations where the participant did not disclose their gender (*n* = 3). There was a statistically significant effect of gender on NSE acknowledgment, *F*(2, 681) = 25.99, *p* < 0.001 (Figure 4). Our secondary hypothesis predicted cisgender men would have the lowest degree of NSE acknowledgment, followed by cisgender women and gender minority people. After running pairwise *t*-tests using Bonferroni corrections, we found that our hypothesis was supported. Specifically, cisgender men (*M* = 1.74, *SD* = 1.59) had the lowest degree of NSE acknowledgment compared to both cisgender women (*M* = 2.50, *SD* = 1.57, *p* < 0.001) and people with a gender minority identity (*M* = 3.54, *SD* = 0.75, *p* < 0.001). Additionally, people with gender minority identities also acknowledged with significantly greater confidence compared to cisgender women (*p* < 0.001). Our BF estimation (*BF*_10_ > 100) corroborated our data and strongly favored the alternative hypothesis over the null.

### 3.4. Exploratory Analyses

Research question 1 asks if PF moderates the relationship between AMASA and NSE acknowledgment. Research question 2 asks if PI moderates the relationship between AMASA and NSE acknowledgment. To explore these research questions, we conducted multiple linear regression models. There was no statistically significant interaction in either model (see Table 3 and Table 4); thus, neither research questions 1 nor 2 were supported. BFs revealed that our data strongly supported the null hypothesis for both research questions 1 (*BF*_01_ = 23.48) and 2 (*BF*_01_ = 7.32).

Research question 3 asks if gender identity moderates the relationship between AMASA and acknowledgment (Table 5). To explore this possibility, we conducted a multiple linear regression. We removed participants who did not provide responses to the gender identity question in the demographics (*n* = 3). There were statistically significant differences among the intercepts of NSE acknowledgment. Overall, when compared to cisgender men, the degree of acknowledgment was higher for cisgender women, *b* = 1.06, *p* < 0.001, 95% CI [0.76, 1.36] and for people with a gender minority people, *b* = 1.88, *p* < 0.001, 95% CI [1.24, 2.51]. Cisgender women were significantly lower in their degree of acknowledgment compared to people with a gender minority identity, *b* = −0.82, *p* < 0.001.

The interaction between AMASA and acknowledgment was also statistically significant across gender identity groups. Specifically, the effect of AMASA on acknowledgment was lower in cisgender men compared to cisgender women, *b* = −0.47, *p* < 0.001, 95% CI [−0.74, −0.19]. However, there were no significant differences in the effect of AMASA on acknowledgment when comparing people with a gender minority identity to either cisgender men or women. The results from the BF strongly favored the alternative hypothesis (*BF*_10_ > 100).

### 3.5. Variation Across Recruitment Sources

The present study was appropriately powered and preregistered to test a focused set of hypotheses (Hypotheses 1–4). Although recruitment targets were met and all preregistered analyses were conducted as planned, participants were recruited through multiple avenues. This raises the possibility that demographic variation across recruitment sources may have influenced the observed effects. As shown in Table 6, minor differences in key demographic distributions were evident across samples. While these variations were relatively small, their potential to moderate key findings warrants consideration in interpreting the results.

We assessed whether the recruitment source influenced the primary findings. Although it would have been ideal to model more fine-grained demographic predictors, doing so in the context of an already exploratory analysis would have increased the risk of statistical inference errors. To balance analytic informativeness with statistical caution, we opted to include recruitment sources alone as a predictor in these models. While this approach likely captures some (though not all) of the demographic variability across recruitment sources, it reduces the risk of overfitting and inflated Type I error rates. Accordingly, we reanalyzed the data for Hypotheses 1–4 using two categorical indicator (dummy) variables to represent participants recruited via the college sample and the snowball sample.

Participants from the Prolific sample served as the reference category (i.e., the intercept). This strategy allowed us to examine whether the recruitment method moderated the hypothesized effects. Participants from the college sample reported fewer NSEs overall compared to those in the Prolific group. However, recruitment from the college sample did not moderate the associations tested in Hypotheses 1–3 (i.e., the relationships between AMASA, PF, and PI, respectively), and the same pattern held for participants recruited via the snowball sample. The only exception emerged in the analysis of Hypothesis 4: cisgender women in the college sample reported fewer NSEs than women in the Prolific sample. Aside from this specific interaction, the recruitment source did not appear to influence the effects observed in the primary analyses. For further details, see the supplementary online analysis scripts: https://osf.io/dwm9g/files/osfstorage.

## 4. Discussion

A person’s acknowledgment may be influenced by AMASA (LeMaire et al., 2016; Peterson & Muehlenhard, 2004; Watters & Yalch, 2023; Wilson et al., 2018). However, AMASA alone does not fully account for acknowledgment (Reed et al., 2019; Wilson & Newins, 2019), suggesting the need for further research on potential moderators (Newins et al., 2018). This study expands the sexual violence literature by exploring additional variables, namely PF and PI, as moderators of the AMASA—NSE acknowledgment relationship. Finally, this study also aimed to address the treatment of gender and ambivalent acknowledgment in the literature.

### 4.1. Prevalence Rates

The prevalence rates for NSE acknowledgment in our sample were consistent with the literature (e.g., Lipinski et al., 2021; Kilimnik et al., 2016). Specifically, 96.5% of people in our sample were identified as having an NSE history based on our behavioral measure. However, only 36.4% of them acknowledged their experiences using a sexual violence label (i.e., responded with “definitely yes”), which is considerably lower than those found in similar studies with a range between 49.6 and 82.9% (Kilimnik et al., 2016; Lipinski et al., 2021; Littleton et al., 2017). The current study asked participants whether they would use the sexual violence labels of “sexual assault” or “rape” to describe their NSEs; these labels do not account for other forms of sexual violence. Researchers may consider including items asking whether participants would use the labels “sexual abuse,” “sexual violence,” “unwanted,” and “nonconsensual” to describe their experiences.

### 4.2. Overview of Findings

Some of our results are consistent with those in the literature, while some were unexpected. For example, we found that people with lower AMASA have a higher degree of acknowledgment than those with higher AMASA (Newins et al., 2018; Peterson & Muehlenhard, 2011). Our findings did not support an association between NSE acknowledgment and PF. Additionally, although a negative relationship between PI and acknowledgment was expected, our results showed that higher levels of PI are associated with a higher degree of sexual violence acknowledgment. While AMASA and PI were independently related to acknowledgment, there were no interactions between them.

### 4.3. Acknowledgment and Psychological Flexibility

We anticipated a positive relationship between PF and NSE acknowledgment largely due to PF being characterized by a willingness to sit with painful and uncomfortable internal and external experiences without attempting to alter them or change their occurrence (e.g., Rolfs et al., 2016). Therefore, to acknowledge one’s NSEs using accepted sexual violence labels would be related to one’s willingness to allow these painful experiences to arise without avoiding them. Self-labeling and applying labels to one’s experiences following NSEs have strong impacts on outcomes in line with the internalization of labels as identity (Graham et al., 2021; O’Shea et al., 2024). The sexual violence literature shows that after sex, self- and other-labeling as “victim,” “survivor,” or “other” correspond to identifying with victim suffering or survivor thriving (O’Shea et al., 2024; Thoits, 2011). Further, RMA may impede persons with NSEs from using sexual violence labels (Rousseau et al., 2020). Many suggest people reject socially constructed labels to avoid associated stereotypes, leading to unacknowledged or ambivalently acknowledged status. (O’Shea et al., 2024; Williamson & Serna, 2018). Nonetheless, our case for the relationship between PF and acknowledgment was not supported by these data.

These results suggest acknowledgment may not be a question of a person’s degree of PF, as the labels a person chooses to describe their NSEs may serve different functions for different people. For example, one person may acknowledge their NSEs as a way of taking control of their painful experiences, seeking a way to flourish or move forward, facilitating post-traumatic growth (e.g., Luang, 2023). However, another person may acknowledge their NSEs and find themselves maintaining psychological distress due to expectations assigned to the specific labels being used (O’Shea et al., 2024). These trajectories are even more likely depending on others’ responses to self-disclosure of traumatic experiences, with post-traumatic growth increasing alongside supportive responses to these disclosures (Luang, 2023), whereas unsupportive responses to vulnerability are associated with adverse psychological outcomes (Criddle et al., 2022).

Additionally, the nonsignificant findings for the relationship between PF and acknowledgment may be attributable to the independent relationships they have with psychological distress. For instance, PF has been found to act as a buffer between early life trauma and the development of depressive and PTSD symptoms in adulthood (Richardson & Jost, 2019). However, NSE acknowledgment has been found to be related to more severe psychological outcomes for those who have an NSE history (Cleere & Lynn, 2013; Lipinski et al., 2021; Littleton et al., 2006). It could be that only specific processes of PF (e.g., acceptance) are relevant to specific degrees of sexual violence acknowledgment. Further, it may be worthwhile to re-examine these research questions, treating acknowledgment as an independent variable (with three levels: acknowledged, ambivalent, and unacknowledged) to see if there are differences across categories of acknowledgment on PF. For instance, by treating acknowledgment as a categorical independent variable rather than a continuous dependent variable, we could assess whether people with specific acknowledgment statuses are higher or lower in PF compared to the other statuses. PF has a distinct relationship with acknowledgment in this sample compared to PI, given the related but independent nature of these constructs.

### 4.4. Acknowledgment and Psychological Inflexibility

PI is characterized, in part, by a person’s efforts to avoid, alter, or suppress painful emotions, thoughts, and experiences. Thus, we predicted PI would have a negative relationship with acknowledgment, with people who are lower in PI having a higher degree of acknowledgment than those higher in PI. While it still may be the case that avoidance efforts (i.e., EA) are associated with a lower degree of acknowledgment, this is only one process of six comprising the PI construct. The PI process of fusion, specifically, could be positively associated with the degree of acknowledgment through qualities supporting the internalization of labels. Fusion’s quality of perceiving one’s thoughts as “true” information often produces behavior driven by thought content rather than by direct interaction with real-world contingencies; specifically, when a person assigns a specific sexual violence label to their NSEs, their behavior may begin to align with the associated social expectations of the label (Schnetzer, 2015).

Domains of sexual violence research in other subfields indicate other possible or overlapping influences. Perhaps acknowledgment contributes to a form of self-discrepancy (i.e., when a person feels they have failed to fulfill their hopes and obligations), wherein they may feel like the label associated with their acknowledgment does not align with the hopes they had for their life (Kwok et al., 2016). Event centrality (i.e., the extent to which a traumatic experience is perceived as a core part of someone’s personal identity) has also been linked to PI (Boykin et al., 2020). Further, the PI process of EA has been found to be positively related to event centrality (Bishop et al., 2018). PI processes may also have varying degrees of influence on acknowledgment, as they have been shown to differentially influence a range of other potentially posttraumatic outcomes (e.g., Landi et al., 2022; Erduran Tekin & Şirin, 2023), indicating a need for future research delineating their roles relative to acknowledgment.

### 4.5. Acknowledgment and Gender Identity

As expected, NSE acknowledgment was associated with gender identity, with cisgender men having the lowest degree of acknowledgment, followed by cisgender women, and then people with a gender minority identity. Testing gender identity as a moderator of the relationship between AMASA and acknowledgment yielded mixed results. Specifically, the interactions between gender identity and AMASA on NSE acknowledgment were stronger for cisgender women compared to cisgender men, yet there were no differences when comparing these groups to those with a gender minority identity. However, the BF for the pairwise comparisons of cisgender people to people with a gender minority identity strongly favored the alternative hypothesis over the null.

These results, especially in the context of our other findings, are consistent with the broader literature. Specifically, people with a gender minority identity tend to endorse AMASA at lower rates compared to cisgender women, who, in turn, endorse AMASA at lower rates compared to cisgender men (Newins et al., 2018; Wilson & Newins, 2019). Furthermore, PI has been found to be positively related to minority stress (Weeks et al., 2020), which would be more prevalent in people with a gender minority identity than people with a gender majority identity and more prevalent in cisgender women than in cisgender men. Taken together, this may elucidate our findings suggesting people with a gender minority identity acknowledge their NSEs to a higher degree than cisgender women, followed by cisgender women and then by cisgender men. That said, once a person uses a specific sexual violence label to describe their history of harm, PI appears to have differential impacts on acknowledgment for affected persons and those perpetrating harm. The relationship between PI and acknowledgment may be a primary reason why those who have acknowledged their NSE history experience heightened post-traumatic stress and depressive symptomatology (Cleere & Lynn, 2013; Lipinski et al., 2021).

### 4.6. Limitations

This study addressed multiple important research gaps, including (1) moving beyond a gender binary view of NSE histories, (2) treating acknowledgment as a continuous variable rather than a dichotomous or categorical one, and (3) examining PF and PI as novel moderating variables for NSE acknowledgment. However, despite the many novel approaches and strengths of this study, it was not without its limitations.

A potential limitation of our study may be related to social desirability. Our large number of unacknowledging participants, alongside our high PF sample mean and low AMASA sample mean, may support this critique. Of course, several methods can be used to assess social desirability (see Lanz et al., 2022), and some of these measures were considered for the current study. However, because the use of these methods remains a point of disagreement in the field (e.g., Kelly et al., 2013), we elected not to implement them. Even so, social desirability may prove to be a consistent obstacle to sexual violence research without a clear solution. However, a meta-analysis (Lanz et al., 2022) on social desirability methods reports offering behavior-contingent incentives (i.e., offering rewards and bonuses as incentives when a specific target behavior is demonstrated by a participant in a study) may be an effective way to reduce social desirability responses. These may prove difficult to offer in basic cross-sectional research, especially when examining sensitive topics such as sexual violence. However, this approach may prove effective if conducting longitudinal studies.

Further, our use of multiple recruitment methods may have contributed to some inconsistent data in our sample. For example, Prolific participants may have been older in age and more educated than our SONA sample, which may have contributed to the Prolific sample being higher in acknowledgment compared to the SONA sample. Our findings support this perspective, showing that age and higher education positively correlated with both types of acknowledgment. Finally, the AMASA measure used in this study was revised specifically to address our specific hypotheses and research questions. Thus, the revisions may have affected its psychometric properties. While the revisions were made to ensure our measure was more specific to the types of MASA relative to NSEs, they limited our ability to compare our scores to those found in the literature. As such, future studies may seek to compare the psychometric properties of the AMMSA-R to the AMMSA-21.

### 4.7. Future Directions

There are several directions future studies may consider for examining NSE acknowledgment. Future research may consider including additional options for assessing acknowledgment. While the current study asked participants whether they would use the sexual violence labels of “sexual assault” or “rape” to describe their NSEs, these labels do not account for all other forms of sexual violence (e.g., Peterson & Muehlenhard, 2004, 2011). Researchers may want to consider including items asking whether participants would use the labels “sexual abuse,” “sexual violence,” “unwanted,” and “nonconsensual” to describe their experiences (Sasson & Paul, 2014). Additionally, the inclusion of a qualitative item allowing participants to describe their NSEs using their own preferred language, as well as an item asking participants to describe the reasons they used or did not use specific labels to describe their experiences, would be invaluable in understanding the variance and nuance of acknowledgment (e.g., Peterson & Muehlenhard, 2004, 2011). Potential studies could also examine what specific experiences participants use certain labels for; this could be performed by administering a sexual history survey (e.g., the PRSPS) and asking how they prefer to label each event.

Additionally, treating acknowledgment as an independent variable with three levels (i.e., acknowledged, ambivalent, unacknowledged) in future research could add insight into group differences in clinically intervenable processes like PF, PI, and AMASA. Future research may also want to examine the core processes comprising PI (i.e., fusion, experiential avoidance, self-as-content, lack of contact with the present moment, lack of contact with values, inaction) and PF (i.e., acceptance, defusion, self-as-context, contact with the present moment, contact with chosen values, and committed action) in relation to degree of acknowledgment and AMASA. Further, it may be beneficial to look at the specific types of MASA a person endorses rather than simply looking at the composite scores of AMASA, similar to the methods used by Peterson and Muehlenhard (2004).

Perhaps most importantly, our study did not consider time elapsed since participants’ NSEs. As noted, this has been found to significantly affect NSE acknowledgment (e.g., Fisher et al., 2000, 2010; Littleton et al., 2006; Newins et al., 2021). All of this has led many to argue that NSE acknowledgment may be a temporal process best examined through longitudinal methods (e.g., Fisher et al., 2000; Littleton et al., 2006; Newins et al., 2021). For these reasons, future researchers have been encouraged to measure the time elapsed since the NSE occurred (see Williamson & Serna, 2018). This is particularly relevant because PI has also been found to be a time-dependent process (e.g., Follette et al., 2010). One area researchers may also consider is the use of daily diaries, ecological momentary assessments, or other longitudinal methods, which can be useful to capture fluctuating or time-dependent relationships indicating non-linear recovery trajectories.

## 5. Conclusions

This research was an important addition to the literature on NSE acknowledgment for several reasons. For instance, it deepened our understanding of the acknowledgment process and aided in more diverse representation in research of people who have an NSE history. It would appear as though acknowledgment itself is not a function of a person’s PF. Rather, acknowledgment may serve different functions for different people. Thus, while acknowledgment is important for research and legal settings, it may not be a key target behavior in clinical settings. As such, clinicians may consider facilitating the development of other target behaviors other than acknowledgment to intervene. They may consider intervening in their client’s relationship with their acknowledgment via the reduction in PI and related distress around the topic using Acceptance and Commitment Therapy (Hayes et al., 1999, 2012). An additional therapeutic target may include a client’s skills related to healthy interpersonal relationships, appropriate vulnerability, and self-disclosure, such as with Functional Analytic Psychotherapy (Follette et al., 2010). Furthermore, researchers may want to shift their focus away from trying to understand why a person may or may not acknowledge their NSE histories and onto people’s specific relationships to their acknowledgment. Therefore, we can better understand how to treat people in clinical settings regarding these behaviors. Sexual violence continues to be a serious problem in the United States (RAINN, n.d.); it is our hope that this research informs applied research for the development and refinement of effective, therapeutic interventions for those who have experienced sexual harm.

## Figures and Tables

**Table 1:** Participant demographic characteristics.

Characteristic	Study Sample (*n* = 697)	Full Sample (*n* = 722)
Age *M* (*SD*)	31.96 (12.26)	31.99 (12.24)
**Gender Identity *n* (%)**		
Man (cis or trans)	136 (19.5%)	154 (21.3%)
Woman (cis or trans)	507 (72.7%)	514 (71.2%)
Gender minority (gender fluid, genderqueer, agender, gender nonbinary)	44 (6.1%)	44 (6.1%)
Unsure	6 (0.9%)	6 (0.8%)
**Sexual Identity *n* (%)**		
Sexual minority (asexual, bisexual or pansexual, gay or lesbian, queer)	205 (29.4%)	208 (28.9%)
Straight/heterosexual	465 (66.7%	487 (67.5%)
Unsure	14 (2.0%)	14 (1.9%)
**Race/Ethnic Identity *n* (%)**		
White/European American	461 (66.1%)	477 (66.1%)
Black/African American	118 (16.9%)	122 (16.9%)
Latin/a/o/x	33 (4.7%)	34 (4.7%)
Asian/Asian American	25 (3.6%)	28 (3.9%)
Indigenous Tribes/First Peoples/Native American	8 (1.1%)	8 (1.1%)
Native Hawaiian or Pacific Islander	2 (0.3%)	2 (0.3%)
More than one race/mixed race	47 (6.7%)	48 (6.6%)
**Level of Education *n* (%)**		
Primary school (elementary)	4 (0.6%)	4 (0.6%)
Secondary school (high school)	138 (19.8%)	142 (19.7%)
Some college or in college	271 (38.9%)	281 (38.9%)
Bachelor’s degree	190 (27.3%)	197 (27.3%)
Graduate degree or in graduate school	79 (11.3%)	83 (11.5%)
Post-graduate degree	7 (1.0%)	7 (1.0%)

Note. Participant ages ranged from 18 to 72 years in both samples; one participant selected to self-describe their gender identity: demiman; several participants (*n* = 10) selected to self-describe their sexual identity: demisexual (*n* = 4), ambisexual (*n* = 1), heteroflexible (*n* = 2), Uranic (*n* = 1), male (*n* = 1), non-label (*n* = 1); several participants (*n* = 6) selected to self-describe their level of education: technical degrees (*n* = 2), trade school (*n* = 2), vocational school (*n* = 2); three participants declined to respond to the gender identity, sexual identity and race/ethnic identity questions; two participants declined to respond to the education question.

**Table 2:** Means, standard deviations, and correlation coefficients between all continuous variables (*N* = 697).

Variable	1	2	3	4	5	6
1. Nonconsensual sexual experience acknowledgment ^1^	-	-	-	-	-	-
2. Acceptance of myths about sexual aggression (AMMSA-R)	−0.17 **	-	-	-	-	-
3. Psychological flexibility (MPFI, Psychological Flexibility)	−0.02	0.21 **	-	-	-	-
4. Psychological inflexibility (MPFI, Psychological Inflexibility)	0.14 **	−0.07 *	−0.44 **	-	-	-
5. Level of Education	0.04	−0.13 **	0.03	−0.09 *	-	-
6. Age	0.08 **	−0.11 **	0.12 **	−0.27 **	0.28 **	-
*M*	2.33	49.45	3.71	2.98	3.37	31.99
*SD*	1.61	18.24	0.85	0.95	1.11	12.24

Note. * *p* < 0.05. ** *p* < 0.01, AMMSA-R = acceptance of modern myths about sexual aggression, revised; MPFI = Multidimensional Psychological Flexibility Inventory; ^1^ because these data were not normally distributed, we ran Kendall’s *tau* to test correlations between these variables; Pearson’s *r* is reported for all other correlations.

**Table 3:** Regression coefficients for main effects and interactions of acceptance of myths about sexual aggression (AMASA) and psychological flexibility (PF) on acknowledgment (*N* = 697).

Variable	*B*	*SE*	*t*	*p*	95% CI
**Model 1**					
Intercept	2.34	0.06	39.08	<0.001	[2.22, 2.46]
AMASA	−0.35	0.06	−5.84	<0.001	[−0.47, −0.23]
PF	0.0001	0.06	0.002	0.99	[−0.12, 0.12]
AMASA × PF	−0.03	0.06	−0.49	0.63	[−0.14, 0.08]
*R* ^2^	0.05				
*BF*_01_	23.85				
**Model 2**					
Intercept	2.37	0.06	40.06	<0.001	[2.25, 2.48]
AMASA	−0.49	0.06	−7.85	<0.001	[−0.61, −0.37]
PF	0.04	0.06	0.58	0.56	[−0.09, 0.16]
AMASA × PF	−0.03	0.06	−0.47	0.64	[−0.16, 0.10]
*R* ^2^	0.08				
*BF*_01_	23.48				

Note. CI = confidence interval; we examined the main effects and interactions of AMASA and PF on acknowledgment; AMASA and PF were the independent variables, and acknowledgment was the dependent variable; multivariate outliers (*n* = 35) were removed in model 2 using Cook’s *D*.

**Table 4:** Regression coefficients for main effects and interactions of AMASA and psychological inflexibility (PI) on acknowledgment (*N* = 697).

Variable	*b*	*SE*	*t*	*p*	95% CI
**Model 1**					
Intercept	2.34	0.06	40.38	<0.001	[2.22, 2.45]
AMASA	−0.33	0.06	−5.78	<0.001	[−0.45, −0.22]
PI	0.29	0.06	5.08	<0.001	[0.18, 0.41]
AMASA × PI	0.04	0.06	0.68	0.498	[−0.07, 0.15]
*R* ^2^	0.08				
*BF_01_*	21.33				
**Model 2**					
Intercept	2.35	0.06	41.42	<0.001	[2.24, 2.47]
AMASA	−0.49	0.06	−8.17	<0.001	[−0.60, −0.37]
PI	0.34	0.06	5.77	<0.001	[0.22, 0.46]
AMASA × PI	0.10	0.06	1.60	.11	[−0.02, 0.22]
*R* ^2^	0.14				
*BF*_01_	7.32				

Note. We examined the main effects and interactions of AMASA and PI on acknowledgment; AMASA and PI were the independent variables, and acknowledgment was the dependent variable; multivariate outliers (*n* = 34) were removed in model 2 using Cook’s *D*.

**Table 5:** Regression coefficients for main effects and interactions of AMASA and gender identity on acknowledgment (*N* = 697).

Variable	*b*	*SE*	*t*	*p*	95% CI
**Model 1**					
Intercept	1.48	0.17	8.58	<0.001	[1.14, 1.82]
AMASA	0.13	0.13	0.04	0.35	[−0.14, 0.39]
Cisgender women	1.07	0.21	5.20	<0.001	[0.66, 1.47]
Gender minority people	1.21	0.35	3.48	<0.001	[0.52, 1.89]
AMASA × Cisgender women	−0.62	0.18	−3.49	<0.001	[−0.97, −0.27]
AMASA × Gender minority people	−0.41	0.29	−1.40	0.16	[−0.98, 0.17]
*R* ^2^	0.15				
*BF*_10_	0.00				
**Model 2**					
Intercept	1.67	0.14	12.35	<0.001	[1.40, 1.93]
AMASA	−0.11	0.12	−0.94	0.35	[−0.34, 0.12]
Cisgender women	0.78	0.15	5.19	<0.001	[0.49, 1.08]
Gender minority people	1.08	0.26	4.16	<0.001	[0.57, 1.60]
AMASA × Cisgender women	−0.47	0.14	−3.40	<0.001	[−0.74, −0.20]
AMASA × Gender minority people	−0.31	0.25	−1.27	0.20	[−0.79, 0.17]
*R* ^2^	0.14				
*BF*_10_	>100				

Note. All gender identity (GI) variables were dummy coded (cisgender men = 0); AMASA and GI were the independent variables, and acknowledgment was the dependent variable; multivariate outliers (*n* = 40) were removed in model 2 using Cook’s *D*.

**Table 6:** Descriptive statistics for key demographics and variables across recruitment methods.

Variable	Prolific (*n* = 503)	Snowball (*n* = 169)	Sona (*n* = 50)
Age *M* (*SD*)	37.09 (10.98)	19.04 (2.08)	24.58 (7.30)
Gender Identity (%)			
Cisgender man	24.3	14.8	6.0
Cisgender woman	68.0	74.6	88.0
Gender minority (transgender, gender fluid, genderqueer, agender, gender nonbinary)	7.8	8.9	6.0
Prefer not to respond	0.0	1.8	0.0
NSE Acknowledgment *M* (*SD*)	2.45 (1.62)	1.86 (1.61)	2.78 (1.25)
AMASA *M* (*SD*)	−0.12 (1.02)	0.44 (0.83)	−0.23 (0.84)
Psychological Flexibility *M* (*SD*)	0.05 (0.99)	−0.09 (0.99)	−0.14 (1.09)
Psychological Inflexibility *M* (*SD*)	−0.09 (1.00)	0.23 (0.96)	0.13 (1.00)

## Data Availability

The original data presented in the study are openly available in the Open Science Framework (OSF) at https://doi.org/10.17605/OSF.IO/36VZF.

## Note

1 The authors of the present study use the same gendered language as the authors being cited unless otherwise noted. This does not reflect the current authors’ commitment to gender inclusivity.

## Supplementary Materials

The following supporting information can be downloaded at: https://www.mdpi.com/article/10.3390/bs15070875/s1 or Open Science Framework (OSF) at https://doi.org/10.17605/OSF.IO/36VZF; Figure S1: Participant flow chart by recruitment method; Table S1: Proportion of label type endorsement; Table S2. Regression coefficients of AMASA on NSE acknowledgment for Hypothesis 1; Table S3: Regression coefficients of PF on NSE acknowledgment for Hypothesis 2; Table S4: Regression coefficients of PI on NSE acknowledgment for Hypothesis 3, project codebook, full cleaned dataset, R code (R Studio file), R code (Word file), all study measures.
