# Supplementary material for: Nonconsensual Sexual Experience Acknowledgment: Exploring the Roles of Gender Identity, Sexual Aggression Myths, and Psychological Inflexibility
Source: Behav Sci (Basel). 2025 Jun 27;15(7):875. doi: 10.3390/bs15070875 (PMC12292596; doi:10.3390/bs15070875)
Supplement: Supplementary file 1 [file behavsci-15-00875-s001.zip › Measures_NSE Acknowledgment_Psyc-Inflex.pdf]

Nonconsensual Sexual Experience Acknowledgment: Exploring the Roles of Gender Identity, Sexual Aggression Myths, and Psychological Inflexibility

Contents

Demographics Questionnaire 2

The Post Refusal Sexual Persistence Scale—Victimization 4

Nonconsensual Sexual Experience Acknowledgment Assessment 6

The Acceptance of Modern Myths about Sexual Aggression, Revised (AMMSA-R) 7

Multidimensional Psychological Flexibility Inventory (MPFI) 9

Narrative Data Validity Check Item 15

Demographics Questionnaire[1]

1. What is your age (in years)? ____________
2. What is your sex at birth?
    a. Male
    b. Female
    c. Intersex
    d. Prefer not to say
    e. Prefer to self-describe/Identity not listed (specify):
3. What is your gender identity?[2]
    a. Man (cis/trans)
    b. Woman (cis/trans)
    c. Gender fluid
    d. Genderqueer
    e. Agender
    f. Nonbinary
    g. Unsure
    h. Prefer not to say
    i. Prefer to self-describe/Identity not listed (specify):
4. What is your sexual orientation?
    a. Asexual
    b. Bisexual or Pansexual
    c. Heterosexual
    d. Gay/Lesbian
    e. Queer
    f. Unsure
    g. Prefer not to say
    h. Prefer to self-describe/Identity not listed (specify):

---

[1] All items on the demographic questionnaire, as well as the associated response options, were randomly presented to participants as to avoid presenting potential identities in a hierarchal manner.
[2] Items "*c*" to "*g*" were collapsed into one gender identity category for this study (i.e., gender minority)

5. What is your race or ethnic identity?
    a. White/European American
    b. Black/African American
    c. Latin/a/o/x
    d. Asian/Asian American
    e. Indigenous Tribes/First People/Native American
    f. Native Hawaiian or Pacific Islander
    g. Mixed race
    h. Prefer not to say
    i. Prefer to self-describe/Racial or ethnic identity not listed (specify):
6. What is your religion or religious philosophy?
    a. Christian (Catholic, Protestant, or any other Christian denominations)
    b. Buddhist
    c. Hindu
    d. Muslim
    e. Jewish
    f. Sikh
    g. Spiritual
    h. Atheist or Agnostic
    i. No religion
    j. Prefer not to say
    k. Prefer to self-describe/Religious identity not listed (specify):
7. What is the highest level of education you have completed?
    a. Primary school
    b. Secondary school
    c. Some college/In college
    d. Bachelor’s degree
    e. Post graduate degree
    f. Post doctoral degree
    g. Prefer not to say
    h. Prefer to self-describe/Level of education not listed (specify):
8. In what year were you born? ____________[3]

[3] Comparing participant responses to items 1 and 8 were used as a data validity check item. If participants' declared age in years matches their declared year of birth, their data were considered valid. If participant age and year of birth do not match, participant data were considered invalid.

The Post Refusal Sexual Persistence Scale—Victimization

Instructions

Since the age of 14, how many times has someone used any of the tactics on the list below to have sexual contact (kissed, fondled, genital touching, oral sex, anal sex, or intercourse) with you after you refused either verbally or nonverbally? Please select the option that best represents the number of times, to the best of your memory, that someone has used a tactic against you. If someone has never used this tactic with you, select “0”. Please answer each question as honestly as possible. How many times?

Response Options

0

1

2—5

6—9

10+

Scale Items

1. Continued to kiss and touch you to arouse you
2. Removed your clothing to arouse you
3. Removed their own clothing to arouse you
4. Tried to talk you into it by repeatedly asking
5. Told you a lie of some kind (told you how much they liked/loved you)
6. Questioned your sexuality (called you frigid/impotent or gay/lesbian)

7. Said they would blackmail you
8. Threatened to harm themselves
9. Used their position of power or authority (a boss, babysitter, teacher)
10. They were an adult at least 5 years older than you and you were under 18
11. They took advantage of the fact that you were drunk or high
12. They gave you alcohol or drugs to get you high
13. They blocked your retreat (closed, locked, or stood blocking the door)
14. They used physical restraint to hold you down or sit on you
15. They tied you up
16. They threatened to physically harm you
17. They physically harmed you (e.g., hit, slapped, or bit you, etc.)
18. They threatened you with a weapon
19. They threatened to break up with you
20. They physically forced you to touch them
21. They got angry with you
22. They guilted you or sulked
23. They threatened to harm someone you are close to
24. They acted together with another person (or people) to overwhelm you

Nonconsensual Sexual Experience Acknowledgment Assessment

Instructions

Please select how much you agree with the following statements:

Response Options

Definitely not

Probably not

Might or might not

Probably yes

Definitely yes

Assessment Items

1. Do you think, since age 14, that you may have ever been sexually assaulted?
2. Do you think, since age 14, that you may have ever been raped?

The Acceptance of Modern Myths about Sexual Aggression, Revised (AMMSA-R)

Instructions

Please rate the extent to which you agree with the following statements using the scale below. Please try to be completely honest.

Response Options

1 = Totally disagree

2 = Disagree

3 = Somewhat disagree

4 = Neither agree nor disagree

5 = Somewhat agree

6 = Agree

7 = Totally agree

Scale Items

1. Many people easily confuse well-intentioned gestures with sexual harassment, aggression, assault, or other forms of sexual violence.
2. People often accuse others of rape or sexual assault in order to hurt them.
3. People often accuse their romantic partner (e.g., husband, wife, boy/girlfriend) of rape or sexual assault to retaliate for a failed relationship.
4. People often accuse more prominent men of rape in order to further their own career.
5. In a custody battle, people often allege that their ex-partner has been sexually violent or aggressive.
6. Men must always be on guard so as not to be accused of sexual violence or aggression.
7. People who have emotional problems often claim that they have been raped.

8. These days, a large number of rape accusations are false.

9. Some women really enjoy playing the victim of rape.

10. Many people like to submit to the sexual wishes of a sex partner.

11. These days, people who share revealing photos make themselves sex objects.

12. When someone plays hard to get, it doesn’t mean they don’t want sex.

13. Good-looking people run a greater risk of becoming victims of sexual violence.

14. In the event of an actual rape, the victim always tries to resist.

15. Most rape victims have taken the risk of walking through dark alleys at night.

16. A man can only be raped by another man.

Multidimensional Psychological Flexibility Inventory (MPFI)

Instructions

Please select how much you agree with each statement as it relates o you over the last two weeks.

Response Options

Never true

Rarely true

Occasionally true

Often true

Very often true

Always true

Inventory Statements (Separated by Subscales)[4]

Flexibility Subscales

Acceptance

1. I was receptive to observing unpleasant thoughts and feelings without interfering with them.
2. I tried to make peace with my negative thoughts and feelings rather than resisting them.
3. I made room to fully experience negative thoughts and emotions, breathing them in rather than pushing them away.
4. When I had an upsetting thought or emotion, I tried to give it space rather than ignoring it.
5. I opened myself to all of my feelings, the good and the bad

[4] When we present the scale to participants, we do not show them the titles of the subscales. Those are only included in this document in the interest of clarity.

Present Moment Awareness

6. I was attentive and aware of my emotions
7. I was in tune with my thoughts and feelings from moment to moment
8. I paid close attention to what I was thinking and feeling
9. I was in touch with the ebb and flow of my thoughts and feelings
10. I strived to remain mindful and aware of my own thoughts and emotions

Self as Context

11. Even when I felt hurt or upset, I tried to maintain a broader perspective
12. I carried myself through tough moments by seeing my life from a larger viewpoint
13. I tried to keep perspective even when life knocked me down
14. When I was scared or afraid, I still tried to see the larger picture
15. When something painful happened, I tried to take a balanced view of the situation

Cognitive Defusion

16. I was able to let negative feelings come and go without getting caught up in them
17. When I was upset, I was able to let those negative feelings pass through me without clinging to them
18. When I was scared or afraid, I was able to gently experience those feelings, allowing them to pass
19. I was able to step back and notice negative thoughts and feelings without reacting to them
20. In tough situations, I was able to notice my thoughts and feelings without getting overwhelmed by them

Values

21. I was very in-touch with what is important to me and my life
22. I stuck to my deeper priorities in life
23. I tried to connect with what is truly important to me on a daily basis
24. Even when it meant making tough choices, I still tried to prioritize the things that were important to me
25. My deeper values consistently gave direction to my life

Committed Action

26. Even when I stumbled in my efforts, I didn't quit working toward what is important
27. Even when times got tough, I was still able to take steps toward what I value in life
28. Even when life got stressful and hectic, I still worked toward things that were important to me
29. I didn't let set-backs slow me down in taking action toward what I really want in life
30. I didn't let my own fears and doubts get in the way of taking action toward my goals

Psychological Inflexibility

Experiential Avoidance

31. When I had a bad memory, I tried to distract myself to make it go away
32. I tried to distract myself when I felt unpleasant emotions
33. When unpleasant memories came to me, I tried to put them out of my mind
34. When something upsetting came up, I tried very hard to stop thinking about it
35. If there was something I didn't want to think about, I would try many things to get it out of my mind

Lack of Contact with the Present Moment

36. I did most things on "automatic" with little awareness of what I was doing
37. I did most things mindlessly without paying much attention
38. I went though most days on auto-pilot without paying much attention to what I was thinking or feeling
39. I floated though most days without paying much attention
40. Most of the time I was just going through the motions without paying much attention

Self as Content

41. I thought some of my emotions were bad or inappropriate and I shouldn't feel them
42. I criticized myself for having irrational or inappropriate emotions
43. I believed some of my thoughts are abnormal or bad and I shouldn't think that way
44. I told myself that I shouldn't be feeling the way I'm feeling
45. I told myself I shouldn't be thinking the way I was thinking

Cognitive Fusion

46. Negative thoughts and feelings tended to stick with me for a long time
47. Distressing thoughts tended to spin around in my mind like a broken record
48. It was very easy to get trapped into unwanted thoughts and feelings
49. When I had negative thoughts or feelings it was very hard to see past them
50. When something bad happened it was hard for me to stop thinking about it

Lack of Contact with Values

51. My priorities and values often fell by the wayside in my day-to-day life
52. When life got hectic, I often lost touch with the things I value
53. The things that I value the most often fell off my priority list completely
54. I didn’t usually have time to focus on the things that are really important to me
55. When times got tough, it was easy to forget about what I truly value

Inaction

56. Negative feelings often trapped me in inaction
57. Negative feelings easily stalled out my plans
58. Getting upset left me stuck and inactive
59. Negative experiences derailed me from what’s really important
60. Unpleasant thoughts and feelings easily overwhelmed my efforts to deepen my life

<u>Scoring</u>

- **Subscales –** To score the MPFI subscales, you assign responses point values from 1 to 6 (left to right as presented above) and then average the responses across the items of each scale so that higher scores reflect higher levels of the dimension being assessed by each set of items.
- **Global Composites –** The averages of the 6 flexibility subscales can be averaged to create a composite representing global flexibility. Similarly, the averages of the 6 inflexibility subscales can be averaged to create a global inflexibility composite.
- **Shorter Global Composites –** The first two items of each of the flexibility subscales can be averaged to create a shorter 12-item global flexibility composite. Similarly, the first 2

items of each of the inflexibility subscales can be averaged to create a shorter 12-item global inflexibility composite.

Narrative Data Validity Check Item

Instructions

Write your answer in the space provided (open text box provided).

Validity Check Question[5]

1. *True or False?* Yellow is a color.

[5] Participants who respond with any other answer than something objectively related to "*True*," were considered invalid respondents (not case sensitive, misspelled to "*tru*," or the abbreviation "*T*" will be accepted).
