# Supplementary material for: Nonconsensual Sexual Experience Acknowledgment: Exploring the Roles of Gender Identity, Sexual Aggression Myths, and Psychological Inflexibility
Source: Behav Sci (Basel). 2025 Jun 27;15(7):875. doi: 10.3390/bs15070875 (PMC12292596; doi:10.3390/bs15070875)
Supplement: Supplementary file 1 [file behavsci-15-00875-s001.zip › R Code_NSE Acknowledgment_Psyc-Inflex.pdf]

```
###IMPORT DATASET

dat <- read.csv("C:/Users/malvi/Downloads/Thesis Pub.csv")
colnames(dat)

################## STANDARDIZING VARIABLES
################################

dat$AMMSA=scale(dat$AMMSA)
dat$PF_Final=scale(dat$PF_Final)
dat$PI_Final=scale(dat$PI_Final)
dat$EA_Final=scale(dat$EA_Final)

###########EVALUATING MISSING RAPE LABELS FROM SNOWBALL SAMPLE

# Is there a real difference between NSEV_SA and max(NSEV_SA, NSEV_R)?
# Take out the people from the snowball sample who didn't answer NSEV_R
no.snow=dat[-c((722-49):722),]

max.id=ifelse(no.snow$NSEV_SA<no.snow$NSEV_R,
              no.snow$NSEV_R,
              no.snow$NSEV_SA)


victims=rbind(
  table(no.snow$NSEV_SA),
  table(max.id))

x2.test=chisq.test(victims)
x2.test


#### Same thing but with perps

# but only people who endorsed PNSE
pnse.only=subset(no.snow,no.snow$PNSE==1)

max.id=ifelse(pnse.only$PNSE_SA<pnse.only$PNSE_R,
              pnse.only$PNSE_R,
              pnse.only$PNSE_SA)
```

```
perps=rbind(
  table(pnse.only$PNSE_SA),
  table(max.id))

x2.test=chisq.test(perps)
x2.test

##########################################################
#     NSE GROUP HYPOTHESES AND RESEARCH QUESTIONS
##########################################################
################## Individual lm()s with NSEV_ID is DV and the following as IVs...

#CREATE SAMPLE SUBSET

nse.only=subset(dat,dat$NSEV==1)

########## HYPOTHESES 1A: AMMSA PREDICTS NSE ACKNOWLEDGMENT
ammsa.mod=lm(NSEV_ID~AMMSA,nse.only)
summary(ammsa.mod)
(ci <- confint(ammsa.mod))

# Look at outliers
cutoff=4/(nrow(dat)-2) # 4 / (N-k-1)
nse.only$get.out=ifelse(cooks.distance(ammsa.mod)>cutoff,1,0)
ammsa.out.dat=subset(nse.only,nse.only$get.out==0)
ammsa.mod.out=lm(NSEV_ID~AMMSA,ammsa.out.dat)
summary(ammsa.mod.out)

(ci <- confint(ammsa.mod.out))

# Bayes Factor
null.mod=lm(NSEV_ID~1,nse.only)
null.mod.out=lm(NSEV_ID~1,ammsa.out.dat)

bf01=exp((BIC(ammsa.mod)-BIC(null.mod))/2)
bf01 # Null is not supported BF01 = <.001

bf01=exp((BIC(ammsa.mod.out)-BIC(null.mod.out))/2)
```

```
bf01 # < .001

##########  HYPOTHESIS 2A: PF PREDICTS NSE ACKNOWLEDGMENT

pf.mod=lm(NSEV_ID~PF_Final,nse.only)
summary(pf.mod)

(ci <- confint(pf.mod))

# Look at outliers
cutoff=4/(nrow(nse.only)-2) # 4 / (N-k-1)
nse.only$get.out=ifelse(cooks.distance(pf.mod)>cutoff,1,0)
pf.out.dat=subset(nse.only,nse.only$get.out==0)
pf.mod.out=lm(NSEV_ID~PF_Final,pf.out.dat)
summary(pf.mod.out)


(ci <- confint(pf.mod.out))

# BAYES FACTOR

null.mod=lm(NSEV_ID~1,nse.only)
null.mod.out=lm(NSEV_ID~1,pf.out.dat)

bf01=exp((BIC(pf.mod)-BIC(null.mod))/2)
bf01 # Null is very supported BF01 = 17.70

bf01=exp((BIC(pf.mod.out)-BIC(null.mod.out))/2)
bf01 # 17.43

########### HYPOTHESIS 3A:  PI PREDICTS NSE ACKNOWLEDGMENT

pi.mod=lm(NSEV_ID~PI_Final,nse.only)
summary(pi.mod)


(ci <- confint(pi.mod))

# Look at outliers
cutoff=4/(nrow(nse.only)-2) # 4 / (N-k-1)
```

```
nse.only$get.out=ifelse(cooks.distance(pi.mod)>cutoff,1,0)
pi.out.dat=subset(nse.only,nse.only$get.out==0)
pi.mod.out=lm(NSEV_ID~PI_Final,pi.out.dat)
summary(pi.mod.out)

(ci <- confint(pi.mod.out))

# BAYES FACTOR

null.mod=lm(NSEV_ID~1,nse.only)
null.mod.out=lm(NSEV_ID~1,pi.out.dat)

bf01=exp((BIC(pi.mod)-BIC(null.mod))/2)
bf01 # < .001

bf01=exp((BIC(pi.mod.out)-BIC(null.mod.out))/2)
bf01 # < .001

########### HYPOTHESIS 4A:  Gender ID PREDICTS NSE ACKNOWLEDGMENT

#CREATE SUBSET ELIMINATING Ss WHO DID NOT PROVIDE GENDER DATA

no.zeroes=subset(nse.only,nse.only$Gender!=0)

#ANALYSES (ANOVA)

gender.mod=aov(NSEV_ID~factor(Gender),no.zeroes)
summary(gender.mod)

(ci <- confint(gender.mod))

men=mean(subset(no.zeroes,no.zeroes$Gender==1)$NSEV_ID)
women=mean(subset(no.zeroes,no.zeroes$Gender==2)$NSEV_ID)
gender.minority=mean(subset(no.zeroes,no.zeroes$Gender==3)$NSEV_ID)


pairwise.t.test(no.zeroes$NSEV_ID,factor(no.zeroes$Gender),
                p.adjust.method = "bonferroni")
```

```
# Look at outliers
no.zeroes$get.out=ifelse(cooks.distance(gender.mod)>cutoff,1,0)
gender.out.dat=subset(no.zeroes,no.zeroes$get.out==0)
gender.mod.out=aov(NSEV_ID~factor(Gender),gender.out.dat)
summary(gender.mod.out)
pairwise.t.test(gender.out.dat$NSEV_ID,factor(gender.out.dat$Gender),
		p.adjust.method = "bonferroni")

# Examine the sample means with regression outliers removed
aggregate(gender.out.dat$NSEV_ID,list(gender.out.dat$Gender),FUN=mean)
aggregate(gender.out.dat$NSEV_ID,list(gender.out.dat$Gender),FUN=sd)
# Number of people in each gender ID group w/ reg. outliers removed
table(gender.out.dat$Gender)

# BAYES FACTOR

null.mod=lm(NSEV_ID~1,dat)
null.mod.out=lm(NSEV_ID~1,gender.out.dat)

bf01=exp((BIC(gender.mod)-BIC(null.mod))/2)
bf01 #

bf01=exp((BIC(gender.mod.out)-BIC(null.mod.out))/2)
bf01 #


########################################NSE GROUP EXPLORATORY ANALYSES

########## Research Question 1: AMMSA x PF

ammsa.mod=lm(NSEV_ID~AMMSA*PF_Final,dat)
summary(ammsa.mod)

(ci <- confint(ammsa.mod))

# Look at outliers
cutoff=4/(nrow(dat)-2) # 4 / (N-k-1)
dat$get.out=ifelse(cooks.distance(ammsa.mod)>cutoff,1,0)
ammsa.out.dat=subset(dat,dat$get.out==0)
ammsa.mod.out=lm(NSEV_ID~AMMSA*PF_Final,ammsa.out.dat)
```

```
summary(ammsa.mod.out)

(ci <- confint(ammsa.mod.out))

# BAYES FACTOR

null.mod=lm(NSEV_ID~AMMSA+PF_Final,dat)
null.mod.out=lm(NSEV_ID~AMMSA+PF_Final,ammsa.out.dat)

bf01=exp((BIC(ammsa.mod)-BIC(null.mod))/2)
bf01 # BF01 > 23.84512

bf01=exp((BIC(ammsa.mod.out)-BIC(null.mod.out))/2)
bf01

########## Research Question 2: AMMSA x PI##########################

ammsa.mod=lm(NSEV_ID~AMMSA*PI_Final,dat)
summary(ammsa.mod)

(ci <- confint(ammsa.mod))


# Look at outliers
cutoff=4/(nrow(dat)-2) # 4 / (N-k-1)
dat$get.out=ifelse(cooks.distance(ammsa.mod)>cutoff,1,0)
ammsa.out.dat=subset(dat,dat$get.out==0)
ammsa.mod.out=lm(NSEV_ID~AMMSA*PI_Final,ammsa.out.dat)
summary(ammsa.mod.out)

(ci <- confint(ammsa.mod.out))

# Since its not significant,

# BAYES FACTOR

null.mod=lm(NSEV_ID~AMMSA+PI_Final,dat)
null.mod.out=lm(NSEV_ID~AMMSA+PI_Final,ammsa.out.dat)

bf01=exp((BIC(ammsa.mod)-BIC(null.mod))/2)
```

```
bf01 #21.33

bf01=exp((BIC(ammsa.mod.out)-BIC(null.mod.out))/2)
bf01# 7.32


########## Research Question 5: AMMSA x Gender ID############################

#CREATE SUBSET ELIMINATING Ss WHO DID NOT PROVIDE GENDER DATA

no.zeros=subset(dat,dat$Gender!=0)

#ANALYSES (LINEAR REGRESSION)

ammsa.mod=lm(NSEV_ID~AMMSA*factor(Gender),no.zeroes)
summary(ammsa.mod)

(ci <- confint(ammsa.mod))

# contrast women and gender minorities

library(multcomp)
# Template for contrasts
# c(
#     intercept
#   , AMMSA
#   , gender 2
#   , gender 3
#   , ammsa x gender 2
#   , ammsa x gender 3
# )

# comparing women and gender minorities
women.v.gm=matrix(c(0,0,1,-1,0,0),1) # intercepts
summary(glht(ammsa.mod,women.v.gm))
women.v.gm=matrix(c(0,0,0,0,1,-1),1) # interactions/slopes
summary(glht(ammsa.mod,women.v.gm))

(ci <- confint(women.v.gm))
```

```
# Look at outliers
cutoff=4/(nrow(dat)-2) # 4 / (N-k-1)
no.zeros$get.out=ifelse(cooks.distance(ammsa.mod)>cutoff,1,0)
ammsa.out.dat=subset(no.zeros,no.zeros$get.out==0)
ammsa.mod.out=lm(NSEV_ID~AMMSA*factor(Gender),ammsa.out.dat)
summary(ammsa.mod.out)

(ci <- confint(ammsa.mod.out))

# comparing women and gender minorities
women.v.gm=matrix(c(0,0,1,-1,0,0),1) # intercepts
summary(glht(ammsa.mod.out,women.v.gm))
women.v.gm=matrix(c(0,0,0,0,1,-1),1) # interactions/slopes
summary(glht(ammsa.mod.out,women.v.gm))

# BAYES FACTOR

null.mod=lm(NSEV_ID~AMMSA+factor(Gender),no.zeros)
null.mod.out=lm(NSEV_ID~AMMSA+factor(Gender),ammsa.out.dat)

bf01=exp((BIC(ammsa.mod)-BIC(null.mod))/2)
bf01

bf01=exp((BIC(ammsa.mod.out)-BIC(null.mod.out))/2)
bf01 # BF01 > 100



##########################################################
#     PNSE GROUP HYPOTHESES AND RESEARCH QUESTIONS
##########################################################

################## Individual lm()s with PNSE is DV and the following as IVs...

#CREATE SAMPLE DATA SUBSET

pnse.only=subset(dat,dat$PNSE==1)

########## HYPOTHESIS 1B: AMMSA
```

```
ammsa.mod=lm(PNSE_ID~AMMSA,pnse.only)
summary(ammsa.mod)

(ci <- confint(ammsa.mod))

# Look at outliers
cutoff=4/(nrow(pnse.only)-2) # 4 / (N-k-1)
pnse.only$get.out=ifelse(cooks.distance(ammsa.mod)>cutoff,1,0)
ammsa.out.dat=subset(pnse.only,pnse.only$get.out==0)
ammsa.mod.out=lm(PNSE_ID~AMMSA,ammsa.out.dat)
summary(ammsa.mod.out)

(ci <- confint(ammsa.mod.out))

######BAYES FACTOR

null.mod=lm(PNSE_ID~1,pnse.only)
null.mod.out=lm(PNSE_ID~1,ammsa.out.dat)

bf01=exp((BIC(ammsa.mod)-BIC(null.mod))/2)
bf01 # Null is supported BF01 = 1.63

bf01=exp((BIC(ammsa.mod.out)-BIC(null.mod.out))/2)
bf01 # 2.11



##########  HYPOTHESIS 2B: PF##########

pf.mod=lm(PNSE_ID~PF_Final,pnse.only)
summary(pf.mod)

(ci <- confint(pf.mod))

# Look at outliers
cutoff=4/(nrow(pnse.only)-2) # 4 / (N-k-1)
pnse.only$get.out=ifelse(cooks.distance(pf.mod)>cutoff,1,0)
pf.out.dat=subset(pnse.only,pnse.only$get.out==0)
pf.mod.out=lm(PNSE_ID~PF_Final,pf.out.dat)
summary(pf.mod.out)
```

```
(ci <- confint(pf.mod.out))


# BF
null.mod=lm(PNSE_ID~1,pnse.only)
null.mod.out=lm(PNSE_ID~1,pf.out.dat)

bf01=exp((BIC(pf.mod)-BIC(null.mod))/2)
bf01 # Null is very supported BF01 = 13.96

bf01=exp((BIC(pf.mod.out)-BIC(null.mod.out))/2)
bf01 # 9.44


###########   HYPOTHESIS 3B: PI ########################################

pi.mod=lm(PNSE_ID~PI_Final,pnse.only)
summary(pi.mod)

(ci <- confint(pi.mod))

# Look at outliers
cutoff=4/(nrow(pnse.only)-2) # 4 / (N-k-1)
pnse.only$get.out=ifelse(cooks.distance(pi.mod)>cutoff,1,0)
pi.out.dat=subset(pnse.only,pnse.only$get.out==0)
pi.mod.out=lm(PNSE_ID~PI_Final,pi.out.dat)
summary(pi.mod.out)

(ci <- confint(pi.mod.out))

#BF

null.mod=lm(PNSE_ID~1,pnse.only)
null.mod.out=lm(PNSE_ID~1,pi.out.dat)

bf01=exp((BIC(pi.mod)-BIC(null.mod))/2)
bf01 # Null is very supported BF01 = 0.53

bf01=exp((BIC(pi.mod.out)-BIC(null.mod.out))/2)
```

```
bf01 # 0.86


###########   HYPOTHESIS 4B: Gender ID ###############################

#SUBSET WITHOUT THOSE WHO DIDN'T GIVE GENDER DATA

no.zeroes=subset(pnse.only,pnse.only$Gender!=0)

###ANALYSES (ANOVA)

gender.mod=aov(PNSE_ID~factor(Gender),no.zeroes)
summary(gender.mod)

(ci <- confint(gender.mod))

men=mean(subset(no.zeroes,no.zeroes$Gender==1)$PNSE_ID)
women=mean(subset(no.zeroes,no.zeroes$Gender==2)$PNSE_ID)
gender.minority=mean(subset(no.zeroes,no.zeroes$Gender==3)$PNSE_ID)


pairwise.t.test(no.zeroes$PNSE_ID,factor(no.zeroes$Gender),
        p.adjust.method = "bonferroni")

# Look at outliers
no.zeroes$get.out=ifelse(cooks.distance(gender.mod)>cutoff,1,0)
gender.out.dat=subset(no.zeroes,no.zeroes$get.out==0)
gender.mod.out=aov(PNSE_ID~factor(Gender),gender.out.dat)
summary(gender.mod.out)
pairwise.t.test(gender.out.dat$PNSE_ID,factor(gender.out.dat$Gender),
        p.adjust.method = "bonferroni")

# Examine the sample means with regression outliers removed
aggregate(gender.out.dat$PNSE_ID,list(gender.out.dat$Gender),FUN=mean)
aggregate(gender.out.dat$PNSE_ID,list(gender.out.dat$Gender),FUN=sd)

# Number of people in each gender ID group w/ reg. outliers removed
table(gender.out.dat$Gender)

# BAYES FACTOR (comparing cis men vs. cis women only)
```

```
bf.fun=subset(no.zeroes,no.zeroes$Gender!=3)

null.mod=lm(PNSE_ID~1,bf.fun)
alt.mod=lm(PNSE_ID~Gender,bf.fun)
bf01=exp((BIC(alt.mod)-BIC(null.mod))/2)
bf01 #15.93

##############################################
# PNSE GROUP EXPLORATORY ANALYSES
##############################################


########## RESEARCH QUESTION 3: AMMSA x PF #######################

ammsa.mod=lm(PNSE_ID~AMMSA*PF_Final,pnse.only)
summary(ammsa.mod)

(ci <- confint(ammsa.mod))

# Look at outliers
cutoff=4/(nrow(pnse.only)-2) # 4 / (N-k-1)
pnse.only$get.out=ifelse(cooks.distance(ammsa.mod)>cutoff,1,0)
ammsa.out.dat=subset(pnse.only,pnse.only$get.out==0)
ammsa.mod.out=lm(PNSE_ID~AMMSA*PF_Final,ammsa.out.dat)
summary(ammsa.mod.out)

(ci <- confint(ammsa.mod.out))


# BF

null.mod=lm(PNSE_ID~AMMSA+PF_Final,pnse.only)
null.mod.out=lm(PNSE_ID~AMMSA+PF_Final,ammsa.out.dat)

bf01=exp((BIC(ammsa.mod)-BIC(null.mod))/2)
bf01 # BF01 > 23.84512

bf01=exp((BIC(ammsa.mod.out)-BIC(null.mod.out))/2)
bf01
```

```
########## RESEARCH QUESTION 4: AMMSA x PI###########################

ammsa.mod=lm(PNSE_ID~AMMSA*PI_Final,pnse.only)
summary(ammsa.mod)

(ci <- confint(ammsa.mod))

# Look at outliers
cutoff=4/(nrow(pnse.only)-2) # 4 / (N-k-1)
pnse.only$get.out=ifelse(cooks.distance(ammsa.mod)>cutoff,1,0)
ammsa.out.dat=subset(pnse.only,pnse.only$get.out==0)
ammsa.mod.out=lm(PNSE_ID~AMMSA*PI_Final,ammsa.out.dat)
summary(ammsa.mod.out)

(ci <- confint(ammsa.mod.out))

# BF

null.mod=lm(PNSE_ID~AMMSA+PI_Final,pnse.only)
null.mod.out=lm(PNSE_ID~AMMSA+PI_Final,ammsa.out.dat)

bf01=exp((BIC(ammsa.mod)-BIC(null.mod))/2)
bf01

bf01=exp((BIC(ammsa.mod.out)-BIC(null.mod.out))/2)
bf01


########## RESEARCH QUESTION 6: AMMSA x Gender ID ##################

##SAMPLE SUBSET FOR NONRESPONDERS

no.zeros=subset(pnse.only,pnse.only$Gender!=0)

##ANALYSIS (LINEAR REGRESSION)
```

```
ammsa.mod=lm(PNSE_ID~AMMSA*factor(Gender),no.zeroes)
summary(ammsa.mod)

(ci <- confint(ammsa.mod))

# contrast women and gender minorities

library(multcomp)
# Template for contrasts
# c(
#     intercept
#   , AMMSA
#   , gender 2
#   , gender 3
#   , ammsa x gender 2
#   , ammsa x gender 3
# )

# comparing women and gender minorities
women.v.gm=matrix(c(0,0,1,-1,0,0),1) # intercepts
summary(glht(ammsa.mod,women.v.gm))
women.v.gm=matrix(c(0,0,0,0,1,-1),1) # interactions/slopes
summary(glht(ammsa.mod,women.v.gm))

(ci <- confint(women.v.gm))

# Look at outliers
cutoff=4/(nrow(pnse.only)-2) # 4 / (N-k-1)
no.zeros$get.out=ifelse(cooks.distance(ammsa.mod)>cutoff,1,0)
ammsa.out.dat=subset(no.zeros,no.zeros$get.out==0)
ammsa.mod.out=lm(PNSE_ID~AMMSA*factor(Gender),ammsa.out.dat)
summary(ammsa.mod.out)

(ci <- confint(ammsa.mod.out))


# comparing women and gender minorities
women.v.gm=matrix(c(0,0,1,-1,0,0),1) # intercepts
summary(glht(ammsa.mod.out,women.v.gm))
```

```
women.v.gm=matrix(c(0,0,0,0,1,-1),1) # interactions/slopes
summary(glht(ammsa.mod.out,women.v.gm))

# BF

null.mod=lm(PNSE_ID~AMMSA+factor(Gender),no.zeros)
null.mod.out=lm(PNSE_ID~AMMSA+factor(Gender),ammsa.out.dat)

bf01=exp((BIC(ammsa.mod)-BIC(null.mod))/2)
bf01

bf01=exp((BIC(ammsa.mod.out)-BIC(null.mod.out))/2)
bf01 # BF01 > 100



########## RESEARCH QUESTION 7: PF x Gender ID#############

ammsa.mod=lm(PNSE_ID~PF_Final*factor(Gender),no.zeroes)
summary(ammsa.mod)

(ci <- confint(ammsa.mod))

# comparing women and gender minorities
women.v.gm=matrix(c(0,0,1,-1,0,0),1) # intercepts
summary(glht(ammsa.mod,women.v.gm))
women.v.gm=matrix(c(0,0,0,0,1,-1),1) # interactions/slopes
summary(glht(ammsa.mod,women.v.gm))


# Look at outliers
cutoff=4/(nrow(pnse.only)-2) # 4 / (N-k-1)
no.zeros$get.out=ifelse(cooks.distance(ammsa.mod)>cutoff,1,0)
ammsa.out.dat=subset(no.zeros,no.zeros$get.out==0)
ammsa.mod.out=lm(PNSE_ID~PF_Final*factor(Gender),ammsa.out.dat)
summary(ammsa.mod.out)

(ci <- confint(ammsa.mod.out))

# comparing women and gender minorities
```

```
women.v.gm=matrix(c(0,0,1,-1,0,0),1) # intercepts
summary(glht(ammsa.mod.out,women.v.gm))
women.v.gm=matrix(c(0,0,0,0,1,-1),1) # interactions/slopes
summary(glht(ammsa.mod.out,women.v.gm))

# BF

null.mod=lm(PNSE_ID~PF_Final+factor(Gender),no.zeros)
null.mod.out=lm(PNSE_ID~PF_Final+factor(Gender),ammsa.out.dat)

bf01=exp((BIC(ammsa.mod)-BIC(null.mod))/2)
bf01

bf01=exp((BIC(ammsa.mod.out)-BIC(null.mod.out))/2)
bf01 # BF01 > 100




########## RESEARCH QUESTION 8: PI x Gender ID###############

ammsa.mod=lm(PNSE_ID~PI_Final*factor(Gender),no.zeroes)
summary(ammsa.mod)

(ci <- confint(ammsa.mod))

# comparing women and gender minorities
women.v.gm=matrix(c(0,0,1,-1,0,0),1) # intercepts
summary(glht(ammsa.mod,women.v.gm))
women.v.gm=matrix(c(0,0,0,0,1,-1),1) # interactions/slopes
summary(glht(ammsa.mod,women.v.gm))


# Look at outliers
cutoff=4/(nrow(pnse.only)-2) # 4 / (N-k-1)
no.zeros$get.out=ifelse(cooks.distance(ammsa.mod)>cutoff,1,0)
ammsa.out.dat=subset(no.zeros,no.zeros$get.out==0)
ammsa.mod.out=lm(PNSE_ID~PI_Final*factor(Gender),ammsa.out.dat)
summary(ammsa.mod.out)
```

```
(ci <- confint(ammsa.mod.out))

# comparing women and gender minorities
women.v.gm=matrix(c(0,0,1,-1,0,0),1) # intercepts
summary(glht(ammsa.mod.out,women.v.gm))
women.v.gm=matrix(c(0,0,0,0,1,-1),1) # interactions/slopes
summary(glht(ammsa.mod.out,women.v.gm))

# BF
null.mod=lm(PNSE_ID~PI_Final+factor(Gender),no.zeros)
null.mod.out=lm(PNSE_ID~PI_Final+factor(Gender),ammsa.out.dat)

bf01=exp((BIC(ammsa.mod)-BIC(null.mod))/2)
bf01

bf01=exp((BIC(ammsa.mod.out)-BIC(null.mod.out))/2)
bf01



########## RESEARCH QUESTION 9: AMMSA + PF + Gender ID############

ammsa.mod=lm(PNSE_ID~AMMSA*PF_Final*factor(Gender),no.zeroes)
summary(ammsa.mod)

(ci <- confint(ammsa.mod))

# Template for contrasts
# c(
#    intercept
#   , AMMSA
#   , PF_Final
#   , gender 2
#   , gender 3
#   , ammsa x PF_Final
#   , ammsa x gender 2
#   , ammsa x gender 3
#   , PF_Final x gender 2
#   , PF_Final x gender 3
```

```
#   , AMMSA x PF x gender 2
#   , AMMSA x PF x gender 3
# )

# comparing women and gender minorities
women.v.gm=matrix(c(0,0,0,0,0,0,0,0,0,0,1,-1),1) # interactions
summary(glht(ammsa.mod,women.v.gm))
women.v.gm=matrix(c(0,0,1,-1,0,0,0,0,0,0,0,0),1) # intercepts
summary(glht(ammsa.mod,women.v.gm))


# Look at outliers
cutoff=4/(nrow(pnse.only)-2) # 4 / (N-k-1)
no.zeros$get.out=ifelse(cooks.distance(ammsa.mod)>cutoff,1,0)
ammsa.out.dat=subset(no.zeros,no.zeros$get.out==0)
ammsa.mod.out=lm(PNSE_ID~AMMSA*PF_Final*factor(Gender),ammsa.out.dat)
summary(ammsa.mod.out)

(ci <- confint(ammsa.mod.out))

# comparing women and gender minorities
women.v.gm=matrix(c(0,0,0,0,0,0,0,0,0,0,1,-1),1) # interactions
summary(glht(ammsa.mod.out,women.v.gm))
women.v.gm=matrix(c(0,0,1,-1,0,0,0,0,0,0,0,0),1) # intercepts
summary(glht(ammsa.mod.out,women.v.gm))

# BF

null.mod=lm(PNSE_ID~AMMSA+PF_Final+factor(Gender)
        +AMMSA*PF_Final+AMMSA*factor(Gender)+PF_Final*factor(Gender)
        ,no.zeros)
null.mod.out=lm(PNSE_ID~AMMSA+PF_Final+factor(Gender)
          +AMMSA*PF_Final+AMMSA*factor(Gender)+PF_Final*factor(Gender)
          ,ammsa.out.dat)

bf01=exp((BIC(ammsa.mod)-BIC(null.mod))/2)
bf01

bf01=exp((BIC(ammsa.mod.out)-BIC(null.mod.out))/2)
bf01
```

```
########## RESEARCH QUESTION 10: AMMSA + PI + Gender ID##########

ammsa.mod=lm(PNSE_ID~AMMSA*PI_Final*factor(Gender),no.zeroes)
summary(ammsa.mod)

(ci <- confint(ammsa.mod))

# comparing women and gender minorities
women.v.gm=matrix(c(0,0,0,0,0,0,0,0,0,0,1,-1),1) # interactions
summary(glht(ammsa.mod.out,women.v.gm))
women.v.gm=matrix(c(0,0,1,-1,0,0,0,0,0,0,0,0),1) # intercepts
summary(glht(ammsa.mod.out,women.v.gm))

# Look at outliers
cutoff=4/(nrow(dat)-2) # 4 / (N-k-1)
no.zeros$get.out=ifelse(cooks.distance(ammsa.mod)>cutoff,1,0)
ammsa.out.dat=subset(no.zeros,no.zeros$get.out==0)
ammsa.mod.out=lm(PNSE_ID~AMMSA*PI_Final*factor(Gender),ammsa.out.dat)
summary(ammsa.mod.out)

(ci <- confint(ammsa.mod.out))

# comparing women and gender minorities
women.v.gm=matrix(c(0,0,0,0,0,0,0,0,0,0,1,-1),1) # interactions
summary(glht(ammsa.mod.out,women.v.gm))
women.v.gm=matrix(c(0,0,1,-1,0,0,0,0,0,0,0,0),1) # intercepts
summary(glht(ammsa.mod.out,women.v.gm))

# BF
null.mod=lm(PNSE_ID~AMMSA+PI_Final+factor(Gender)
            +AMMSA*PI_Final+AMMSA*factor(Gender)+PI_Final*factor(Gender)
            ,no.zeros)
null.mod.out=lm(PNSE_ID~AMMSA+PI_Final+factor(Gender)
                +AMMSA*PI_Final+AMMSA*factor(Gender)+PI_Final*factor(Gender)
                ,ammsa.out.dat)

bf01=exp((BIC(ammsa.mod)-BIC(null.mod))/2)
```

```
bf01

bf01=exp((BIC(ammsa.mod.out)-BIC(null.mod.out))/2)
bf01



##########################################################
#     DNSE GROUP: RESEARCH QUESTIONS
######################################################


################## Individual lm()s with PNSE is DV and the following as IVs...

#CREATE SAMPLE DATA SUBSET

dnse.only=subset(dat,dat$DNSE==1)


########## RQ11: AMMSA

ammsa.mod=lm(NSEV_ID~AMMSA,dnse.only)
summary(ammsa.mod)

(ci <- confint(ammsa.mod))

# Look at outliers
cutoff=4/(nrow(dnse.only)-2) # 4 / (N-k-1)
dnse.only$get.out=ifelse(cooks.distance(ammsa.mod)>cutoff,1,0)
ammsa.out.dat=subset(dnse.only,dnse.only$get.out==0)
ammsa.mod.out=lm(NSEV_ID~AMMSA,ammsa.out.dat)
summary(ammsa.mod.out)

(ci <- confint(ammsa.mod.out))

# BF

null.mod=lm(NSEV_ID~1,dnse.only)
null.mod.out=lm(NSEV_ID~1,ammsa.out.dat)
```

```
bf01=exp((BIC(ammsa.mod)-BIC(null.mod))/2)
bf01 #  0.003

bf01=exp((BIC(ammsa.mod.out)-BIC(null.mod.out))/2)
bf01 # < .001


##########  RESEARCH QUESTION 12: PF #################################

pf.mod=lm(NSEV_ID~PF_Final,dnse.only)
summary(pf.mod)

(ci <- confint(pf.mod))

# Look at outliers
cutoff=4/(nrow(dnse.only)-2) # 4 / (N-k-1)
dnse.only$get.out=ifelse(cooks.distance(pf.mod)>cutoff,1,0)
pf.out.dat=subset(dnse.only,dnse.only$get.out==0)
pf.mod.out=lm(NSEV_ID~PF_Final,pf.out.dat)
summary(pf.mod.out)

(ci <- confint(pf.mod.out))

# BF

null.mod=lm(NSEV_ID~1,dnse.only)
null.mod.out=lm(NSEV_ID~1,pf.out.dat)

bf01=exp((BIC(pf.mod)-BIC(null.mod))/2)
bf01 #  6.87

bf01=exp((BIC(pf.mod.out)-BIC(null.mod.out))/2)
bf01 # 2.11


########### RESEARCH QUESTION 13:  PI ##################################

pi.mod=lm(NSEV_ID~PI_Final,dnse.only)
summary(pi.mod)
```

```
(ci <- confint(pi.mod))

# Look at outliers
cutoff=4/(nrow(dnse.only)-2) # 4 / (N-k-1)
dnse.only$get.out=ifelse(cooks.distance(pi.mod)>cutoff,1,0)
pi.out.dat=subset(pnse.only,pnse.only$get.out==0)
pi.mod.out=lm(NSEV_ID~PI_Final,pi.out.dat)
summary(pi.mod.out)

(ci <- confint(pi.mod.out))

# BF

null.mod=lm(NSEV_ID~1,dnse.only)
null.mod.out=lm(NSEV_ID~1,pi.out.dat)

bf01=exp((BIC(pi.mod)-BIC(null.mod))/2)
bf01 # 0.01

bf01=exp((BIC(pi.mod.out)-BIC(null.mod.out))/2)
bf01 # Inf




########### RESEARCH QUESTION 14:  Gender ID ##################################

#SUBSET OF NONRESPONDERS

no.zeroes=subset(dnse.only,dnse.only$Gender!=0)

#ANALYSIS (ANOVA)

gender.mod=aov(NSEV_ID~factor(Gender),no.zeroes)
summary(gender.mod)

(ci <- confint(gender.mod))
```

```
men=mean(subset(no.zeroes,no.zeroes$Gender==1)$NSEV_ID)
women=mean(subset(no.zeroes,no.zeroes$Gender==2)$NSEV_ID)
gender.minority=mean(subset(no.zeroes,no.zeroes$Gender==3)$NSEV_ID)


pairwise.t.test(no.zeroes$NSEV_ID,factor(no.zeroes$Gender),
                p.adjust.method = "bonferroni")

# Look at outliers
no.zeroes$get.out=ifelse(cooks.distance(gender.mod)>cutoff,1,0)
gender.out.dat=subset(no.zeroes,no.zeroes$get.out==0)
gender.mod.out=aov(NSEV_ID~factor(Gender),gender.out.dat)
summary(gender.mod.out)
pairwise.t.test(gender.out.dat$NSEV_ID,factor(gender.out.dat$Gender),
                p.adjust.method = "bonferroni")

# Examine the sample means with regression outliers removed
aggregate(gender.out.dat$NSEV_ID,list(gender.out.dat$Gender),FUN=mean)
aggregate(gender.out.dat$NSEV_ID,list(gender.out.dat$Gender),FUN=sd)

# Number of people in each gender ID group w/ reg. outliers removed
table(gender.out.dat$Gender)

# BF

null.mod=lm(NSEV_ID~1,dnse.only)
null.mod.out=lm(NSEV_ID~1,gender.out.dat)

bf01=exp((BIC(gender.mod)-BIC(null.mod))/2)
bf01 # < .001

bf01=exp((BIC(gender.mod.out)-BIC(null.mod.out))/2)
bf01 # < .001

##########RQ15:  AMMSA x PF###############

ammsa.mod=lm(NSEV_ID~AMMSA*PF_Final,pnse.only)
summary(ammsa.mod)

(ci <- confint(ammsa.mod))
```

```
# Look at outliers
cutoff=4/(nrow(pnse.only)-2) # 4 / (N-k-1)
pnse.only$get.out=ifelse(cooks.distance(ammsa.mod)>cutoff,1,0)
ammsa.out.dat=subset(pnse.only,pnse.only$get.out==0)
ammsa.mod.out=lm(NSEV_ID~AMMSA*PF_Final,ammsa.out.dat)
summary(ammsa.mod.out)

(ci <- confint(ammsa.mod.out))

mod1=lm(PNSE_ID~Age+factor(Gender_Full)+factor(Sex_Or)+factor(Race)+factor(Religion)+
Educ,pnse.only)
mod2=lm(PNSE_ID~PF_Final+Age+factor(Gender_Full)+factor(Sex_Or)+factor(Race)+factor(
Religion)+Educ,pnse.only)
anova(mod1,mod2)

# BF

null.mod=lm(NSEV_ID~AMMSA+PF_Final,pnse.only)
null.mod.out=lm(NSEV_ID~AMMSA+PF_Final,ammsa.out.dat)

bf01=exp((BIC(ammsa.mod)-BIC(null.mod))/2)
bf01 # BF01 > 16.92

bf01=exp((BIC(ammsa.mod.out)-BIC(null.mod.out))/2)
bf01 # 14.22



#########RQ16: AMMSA x PI################

ammsa.mod=lm(NSEV_ID~AMMSA*PI_Final,pnse.only)
summary(ammsa.mod)

(ci <- confint(ammsa.mod))

# Look at outliers
cutoff=4/(nrow(pnse.only)-2) # 4 / (N-k-1)
pnse.only$get.out=ifelse(cooks.distance(ammsa.mod)>cutoff,1,0)
ammsa.out.dat=subset(pnse.only,pnse.only$get.out==0)
```

```
ammsa.mod.out=lm(NSEV_ID~AMMSA*PI_Final,ammsa.out.dat)
summary(ammsa.mod.out)

(ci <- confint(ammsa.mod.out))


# BF

null.mod=lm(NSEV_ID~AMMSA+PI_Final,pnse.only)
null.mod.out=lm(NSEV_ID~AMMSA+PI_Final,ammsa.out.dat)

bf01=exp((BIC(ammsa.mod)-BIC(null.mod))/2)
bf01 #16.94

bf01=exp((BIC(ammsa.mod.out)-BIC(null.mod.out))/2)
bf01 #15.56



##########RQ17: AMMSA x Gender ID##########

#SUBSET OF NONRESPONDERS

no.zeros=subset(dnse.only,dnse.only$Gender!=0)

#ANALYSIS (LINEAR REGRESSION)

ammsa.mod=lm(NSEV_ID~AMMSA*factor(Gender),no.zeroes)
summary(ammsa.mod)

(ci <- confint(ammsa.mod))

# contrast women and gender minorities

library(multcomp)
# Template for contrasts
# c(
#     intercept
#   , AMMSA
#   , gender 2
```

```
#   , gender 3
#   , ammsa x gender 2
#   , ammsa x gender 3
# )

# comparing women and gender minorities
women.v.gm=matrix(c(0,0,1,-1,0,0),1) # intercepts
summary(glht(ammsa.mod,women.v.gm))
women.v.gm=matrix(c(0,0,0,0,1,-1),1) # interactions/slopes
summary(glht(ammsa.mod,women.v.gm))

(ci <- confint(women.v.gm))

# Look at outliers
cutoff=4/(nrow(dnse.only)-2) # 4 / (N-k-1)
no.zeros$get.out=ifelse(cooks.distance(ammsa.mod)>cutoff,1,0)
ammsa.out.dat=subset(no.zeros,no.zeros$get.out==0)
ammsa.mod.out=lm(NSEV_ID~AMMSA*factor(Gender),ammsa.out.dat)
summary(ammsa.mod.out)

(ci <- confint(ammsa.mod.out))


# comparing women and gender minorities
women.v.gm=matrix(c(0,0,1,-1,0,0),1) # intercepts
summary(glht(ammsa.mod.out,women.v.gm))
women.v.gm=matrix(c(0,0,0,0,1,-1),1) # interactions/slopes
summary(glht(ammsa.mod.out,women.v.gm))

# BF

null.mod=lm(NSEV_ID~AMMSA+factor(Gender),no.zeros)
null.mod.out=lm(NSEV_ID~AMMSA+factor(Gender),ammsa.out.dat)

bf01=exp((BIC(ammsa.mod)-BIC(null.mod))/2)
bf01

bf01=exp((BIC(ammsa.mod.out)-BIC(null.mod.out))/2)
bf01 # BF01 > 100
```

```
###################DESCRIPTIVES AND DEMOGRAPHICS######################

########### Whole sample demographics
mean(dat$Age) #31.99
sd(dat$Age) #12.24
summary(dat$Age)#18-72
nrow(dat)
table(dat$Race)
table(dat$Sex)
table(dat$Gender_Full)
table(dat$Sex_Or)
table(dat$Religion)
table(dat$Educ)
table(dat$Gender)# 0 = 3, cismale = 150; cisfemale = 512; gender minority = 57

########### PNSE only demographics
pnse.only=subset(dat,dat$PNSE==1)
nrow(pnse.only)
no.snow.pnes.only=subset(pnse.only,pnse.only$Sample!=3)
nrow(no.snow.pnes.only)
no.zeroes.pnse.only=subset(pnse.only,pnse.only$Gender!=0)
nrow(no.zeroes.pnes.only)
mean(pnse.only$Age) #33.79
sd(pnse.only$Age) # 12.34
summary(pnse.only$Age) #18-70
table(pnse.only$Race)
table(pnse.only$Sex)
table(pnse.only$Gender_Full)
table(pnse.only$Sex_Or)
table(pnse.only$Religion)
table(pnse.only$Educ)
table(pnse.only$Gender) #0 = 2; cismale = 94; cisfemale = 182; gender minority = 27
no.snow.pnes.only=subset(pnse.only,pnse.only$Sample!=3)

############ NSE only demographics
nse.only=subset(dat,dat$NSEV==1)
nrow(nse.only)
no.snow.nse.only=subset(nse.only,nse.only$Sample!=3)
nrow(no.snow.nse.only)
no.zeroes.nse.only=subset(nse.only,nse.only$Gender!=0)
```

```
nrow(no.zeroes.nse.only)
mean(nse.only$Age) #31.96
sd(nse.only$Age) # 12.26
summary(nse.only$Age) #18-72
table(nse.only$Race)
table(nse.only$Sex)
table(nse.only$Gender_Full)
table(nse.only$Sex_Or)
table(nse.only$Educ)
table(nse.only$Gender) #0 = 3; cism = 132; cisw = 506; gm = 56

################# DNSE only demographics
dnse.only=subset(dat,dat$DNSE==1)
nrow(dnse.only)
no.snow.dnse.only=subset(dnse.only,dnse.only$Sample!=3)
nrow(no.snow.dnse.only)
no.zeroes.dnse.only=subset(dnse.only,dnse.only$Gender!=0)
nrow(no.zeroes.dnse.only)
mean(dnse.only$Age) #33.86
sd(dnse.only$Age) # 12.41
summary(dnse.only$Age) #18-70
table(dnse.only$Race)
table(dnse.only$Sex)
table(dnse.only$Gender_Full)
table(dnse.only$Sex_Or)
table(dnse.only$Educ)
table(dnse.only$Gender)# 0 = 2; cism = 76; cisw = 176; gm = 26

###################PREVALENCE OF NSE  LABEL TYPE IN NSE HISTORY

# How many people have a 4 on NSEV_R
# Take out people from the snowball sample first
no.snow=nse.only[-c(673:722),]

# Number of no snows = nrow(no.snow) = 672
sum(ifelse(no.snow.nse.only$NSEV_R==4,1,0)) # 125

# How many of whole sample had 4 on NSEV_SA
sum(ifelse(nse.only$NSEV_SA==4,1,0)) # 232
```

```
# How many of no snows had 0 for NSEV_R
sum(ifelse(no.snow.nse.only$NSEV_R==0,1,0)) # 346
# And for NSEV_SA
sum(ifelse(nse.only$NSEV_SA==0,1,0)) # 177

# How many of no snows had 1 for NSEV_R
sum(ifelse(no.snow.nse.only$NSEV_R==1,1,0)) # 57
# And for NSEV_SA
sum(ifelse(nse.only$NSEV_SA==1,1,0)) # 80

# How many of no snows had 2 for NSEV_R
sum(ifelse(no.snow.nse.only$NSEV_R==2,1,0)) # 65
# And for NSEV_SA
sum(ifelse(nse.only$NSEV_SA==2,1,0)) # 69

# How many of no snows had 3 for NSEV_R
sum(ifelse(no.snow.nse.only$NSEV_R==3,1,0)) # 54
# And for NSEV_SA
sum(ifelse(nse.only$NSEV_SA==3,1,0)) # 139

# How many of no snows had 1-3 for NSEV_R
sum(ifelse(no.snow.nse.only$NSEV_R!=0 & no.snow.nse.only$NSEV_R!=4,1,0)) # 176
# And for NSEV_SA
sum(ifelse(nse.only$NSEV_SA!=0 & nse.only$NSEV_SA!=4,1,0)) # 288

####################PREVALENCE OF PNSE LABEL TYPE IN PNSE HISTORY

# How many people have a 4 on PNSE_R
# Take out people from the snowball sample first
no.snow=pnse.only[-c(673:722),]
# Number of no snows = nrow(no.snow) =
sum(ifelse(no.snow.pnes.only$PNSE_R==4,1,0)) # 3

# How many of whole sample had 4 on PNSE_SA
sum(ifelse(pnse.only$PNSE_SA==4,1,0)) # 10


# How many of no snows had 0 for PNSE_R
sum(ifelse(no.snow.pnes.only$PNSE_R==0,1,0)) # 254
```

```
# And for PNSE_SA
sum(ifelse(pnse.only$PNSE_SA==0,1,0)) # 197

# How many of no snows had 1 for pnse_R
sum(ifelse(no.snow.pnes.only$PNSE_R==1,1,0)) # 24
# And for PNSE_SA
sum(ifelse(pnse.only$PNSE_SA==1,1,0)) # 58

# How many of no snows had 2 for PNSE_R
sum(ifelse(no.snow.pnes.only$PNSE_R==2,1,0)) # 4
# And for PNSE_SA
sum(ifelse(pnse.only$PNSE_SA==2,1,0)) # 24

# How many of no snows had 3 for PNSE_R
sum(ifelse(no.snow.pnes.only$PNSE_R==3,1,0)) # 2
# And for NSEV_SA
sum(ifelse(pnse.only$PNSE_SA==3,1,0)) # 16

# How many of no snows had 1-3 for PNSE_R
sum(ifelse(no.snow.pnes.only$PNSE_R!=0 & no.snow.pnes.only$PNSE_R!=4,1,0)) # 30
# And for PNSE_SA
sum(ifelse(pnse.only$PNSE_SA!=0 & pnse.only$PNSE_SA!=4,1,0)) # 98

###################PREVALENCE OF NSE LABEL TYPE IN DNSE HISTORY

# How many people have a 4 on NSEV_R
# Take out people from the snowball sample first
no.snow=dnse.only[-c(673:722),]
# Number of no snows = nrow(no.snow) =
sum(ifelse(no.snow.dnse.only$NSEV_R==4,1,0)) # 51

# How many of whole sample had 4 on NSEV_SA
sum(ifelse(dnse.only$NSEV_SA==4,1,0)) # 87


# How many of no snows had 0 for NSEV_R
sum(ifelse(no.snow.dnse.only$NSEV_R==0,1,0)) # 133
# And for PNSE_SA
sum(ifelse(dnse.only$NSEV_SA==0,1,0)) # 69
```

```
# How many of no snows had 1 for NSEV_R
sum(ifelse(no.snow.dnse.only$NSEV_R==1,1,0)) # 31
# And for PNSE_SA
sum(ifelse(dnse.only$NSEV_SA==1,1,0)) # 30

# How many of no snows had 2 for NSEV_R
sum(ifelse(no.snow.dnse.only$NSEV_R==2,1,0)) # 26
# And for NSEV_SA
sum(ifelse(dnse.only$NSEV_SA==2,1,0)) # 33

# How many of no snows had 3 for NSEV_R
sum(ifelse(no.snow.dnse.only$NSEV_R==3,1,0)) # 21
# And for NSEV_SA
sum(ifelse(dnse.only$NSEV_SA==3,1,0)) # 61

# How many of no snows had 1-3 for NSEV_R
sum(ifelse(no.snow.dnse.only$NSEV_R!=0 & no.snow.dnse.only$NSEV_R!=4,1,0)) # 78
# And for NSEV_SA
sum(ifelse(dnse.only$NSEV_SA!=0 & dnse.only$NSEV_SA!=4,1,0)) # 124

####################PREVALENCE OF NSE ACKNOWLEDGEMENT IN NSE GROUP

# How many people have a 4 on NSEV_ID


# How many of whole sample had 4 on NSEV_ID
sum(ifelse(nse.only$NSEV_ID==4,1,0)) # 260


# How many of no snows had 0 for NSEV_ID
sum(ifelse(nse.only$NSEV_ID==0,1,0)) # 156

# How many of no snows had 1 for NSEV_ID
sum(ifelse(nse.only$NSEV_ID==1,1,0)) # 75

# How many of no snows had 2 for NSEV_ID
sum(ifelse(nse.only$NSEV_ID==2,1,0)) # 70

# How many of no snows had 3 for NSEV_ID
sum(ifelse(nse.only$NSEV_ID==3,1,0)) # 136
```

```
# How many of no snows had 1-3 for NSEV_ID
sum(ifelse(nse.only$NSEV_ID!=0 & nse.only$NSEV_ID!=4,1,0)) # 281


###################PREVALENCE OF PNSE ACKNOWLEDGMENT IN PNSE GROUP

# How many people have a 4 on PNSE_

# How many of whole sample had 4 on PNSE_ID
sum(ifelse(pnse.only$PNSE_ID==4,1,0)) # 10


# How many of no snows had 0 for PNSE_ID
sum(ifelse(pnse.only$PNSE_ID==0,1,0)) # 196

# How many of no snows had 1 for PNSE_ID
sum(ifelse(pnse.only$PNSE_ID==1,1,0)) # 58

# How many of no snows had 2 for PNSE_ID
sum(ifelse(pnse.only$PNSE_ID==2,1,0)) # 25

# How many of no snows had 3 for PNSE_ID
sum(ifelse(pnse.only$PNSE_ID==3,1,0)) #16

# How many of no snows had 1-3 for PNSE_ID
sum(ifelse(pnse.only$PNSE_ID!=0 & pnse.only$PNSE_ID!=4,1,0)) # 99

###################PREVALENCE OF NSE ACKNOWLEDGEMENT IN DNSE GROUP

# How many people have a 4 on NSEV_ID
sum(ifelse(dnse.only$NSEV_ID==4,1,0)) # 101


# How many of no snows had 0 for NSEV_ID
sum(ifelse(dnse.only$NSEV_ID==0,1,0)) # 60

# How many of no snows had 1 for NSEV_ID
sum(ifelse(dnse.only$NSEV_ID==1,1,0)) # 31
```

```
# How many of no snows had 2 for NSEV_ID
sum(ifelse(dnse.only$NSEV_ID==2,1,0)) # 32

# How many of no snows had 3 for NSEV_ID
sum(ifelse(dnse.only$NSEV_ID==3,1,0)) # 56

# How many of no snows had 1-3 for NSEV_ID
sum(ifelse(dnse.only$NSEV_ID!=0 & dnse.only$NSEV_ID!=4,1,0)) # 119



##########SPREAD, CENTRAL TENDENCY, and CROHNBACH'S ALPHA############

#AMMSA DATASET

AMMSA_RAW = read.csv("C:/Users/malvi/Desktop/Thesis/Descriptives/AMMSA_RAW - AMMSA_RAW.csv")
read.csv("C:/Users/malvi/Desktop/Thesis/Descriptives/AMMSA_RAW - AMMSA_RAW.csv")
colnames(AMMSA_RAW)


#######FULL SAMPLE

#AMMSA

mean(dat$AMMSA) # 49.45
sd(dat$AMMSA) #18.24
summary(dat$AMMSA)# 16.00-98.00

psych::alpha(AMMSA_RAW)[1]

#NSE ACKNOWLEDGMENT

mean(dat$NSEV_ID) # 2.33
sd(dat$NSEV_ID) # 1.61
summary(dat$NSEV_ID)# 0.00-4.00

#PNSE ACKNOWLEDGMENT

mean(pnse.only$PNSE_ID) # 0.64
```

```
sd(pnse.only$PNSE_ID) # 1.05
summary(pnse.only$PNSE_ID)# 0.00-4.00

#MPFI-PF

MPFI.PF_RAW = read.csv("C:/Users/malvi/Desktop/Thesis/Descriptives/MPFI.PF_RAW -
MPFI.PF_RAW.csv")
colnames(MPFI.PF_RAW)

mean(dat$PF_Final) # 3.71
sd(dat$PF_Final) # 0.85
summary(dat$PF_Final)# 1.00-6.00
psych::alpha(MPFI.PF_RAW)[1]

#MPFI-PI

MPFI.PI_RAW = read.csv("C:/Users/malvi/Desktop/Thesis/Descriptives/MPFI.PI_RAW -
MPFI.PI_RAW.csv")
colnames(MPFI.PI_RAW)

mean(dat$PI_Final) # 2.98
sd(dat$PI_Final) # 0.95
summary(dat$PI_Final)# 1.00-5.77
psych::alpha(MPFI.PI_RAW)[1]

#EDUCATION

mean(dat$Educ) #3.37
sd(dat$Educ) #1.11
summary(dat$Educ)

# ACKNOWLEDGMENT

ID_RAW <- read.csv("C:/Users/malvi/Downloads/ID_RAW.csv")
colnames(ID_RAW)
psych::alpha(ID_RAW)[1]#0.67


#############CORRELATIONS MATRIX##################################
```

```
#VARIABLE HISTOGRAMS

hist(pnse.only$PNSE_ID) #nonnormally distributed
hist(dat$NSEV_ID) #nonnormally distributed
hist(dat$AMMSA) #normally distributed
hist(dat$PF_Final) #normally distributed
hist(dat$PI_Final) #normally distributed
hist(dat$Educ) #normally distributed

#MATRIX

matrix.dat=dat[,-c(23:26)]
cor(matrix.dat)

#CORRELATIONS (PEARSON'S R)

cor.test(dat$NSEV_ID,dat$PNSE_ID)
cor.test(dat$NSEV_ID,dat$AMMSA)
cor.test(dat$NSEV_ID,dat$PF_Final)
cor.test(dat$NSEV_ID,dat$PI_Final)
cor.test(dat$NSEV_ID,dat$Educ)
cor.test(dat$NSEV_ID,dat$Age)

cor.test(pnse.only$PNSE_ID,dat$AMMSA)
cor.test(pnse.only$PNSE_ID,dat$PF_Final)
cor.test(pnse.only$PNSE_ID,dat$PI_Final)
cor.test(pnse.only$PNSE_ID,dat$Educ)
cor.test(pnse.only$PNSE_ID,dat$Age)

cor.test(dat$AMMSA,dat$PF_Final)
cor.test(dat$AMMSA,dat$PI_Final)
cor.test(dat$AMMSA,dat$Educ)
cor.test(dat$AMMSA,dat$Age)


cor.test(dat$PF_Final,dat$PI_Final)
cor.test(dat$PF_Final,dat$Educ)
cor.test(dat$PF_Final,dat$Age)

cor.test(dat$PI_Final,dat$Educ)
```

```
cor.test(dat$PI_Final,dat$Age)

cor.test(dat$Educ,dat$Age)

#CORRELATIONS (KENDALLS TAU)

cor.test(dat$NSEV_ID, dat$PNSE_ID,method="kendall")
cor.test(dat$NSEV_ID, dat$AMMSA,method="kendall")
cor.test(dat$NSEV_ID, dat$PF_Final,method="kendall")
cor.test(dat$NSEV_ID, dat$PI_Final,method="kendall")
cor.test(dat$NSEV_ID, dat$Educ,method="kendall")
cor.test(dat$NSEV_ID, dat$Age,method="kendall")

cor.test(pnse.only$PNSE_ID, pnse.only$AMMSA,method="kendall")
cor.test(pnse.only$PNSE_ID, pnse.only$PF_Final,method="kendall")
cor.test(pnse.only$PNSE_ID, pnse.only$PI_Final,method="kendall")
cor.test(pnse.only$PNSE_ID, pnse.only$Educ,method="kendall")
cor.test(pnse.only$PNSE_ID, pnse.only$Age,method="kendall")

#############################DIFFERENCES BETWEEN SAMPLES###############

# Differ by recruitement method?
summary(aov(dat$NSEV_ID~factor(dat$Sample)))

library(ggplot2)
library(ggpubr)

ggbarplot(dat,x="Sample",y="NSEV_ID",
    add="mean_se", ylab="NSEV_ID",
    xlab="Recruited from")

pairwise.t.test(dat$NSEV_ID,dat$Sample,p.adjust.method="bonf")


# Does PNSE ~ AMMSA for groups 1 + 3, but not 2?
pnse.only$sona=ifelse(pnse.only$Sample==2,1,0)
summary(lm(PNSE_ID~AMMSA*factor(sona),pnse.only))


# Does NSEV_ID ~ PF for groups 1 + 3, but not 2?
```

```
dat$sona=ifelse(dat$Sample==2,1,0)
summary(lm(NSEV_ID~PF_Final*factor(sona),dat))

# Does NSEV_ID ~ PF for group 1 only
prolific=subset(dat,dat$Sample==1)
summary(lm(NSEV_ID~PF_Final,prolific))
```
