# Supplementary material for: Nonconsensual Sexual Experience Acknowledgment: Exploring the Roles of Gender Identity, Sexual Aggression Myths, and Psychological Inflexibility
Source: Behav Sci (Basel). 2025 Jun 27;15(7):875. doi: 10.3390/bs15070875 (PMC12292596; doi:10.3390/bs15070875)
Supplement: Supplementary file 1 [file behavsci-15-00875-s001.zip › Supplementary-Figures-Tables_NSE Acknowledgment_Psyc-Inflex.pdf]

Nonconsensual Sexual Experience Acknowledgment: Exploring the Roles of Gender Identity, Sexual Aggression Myths, and Psychological Inflexibility

Wesley Malvini, Jessica M. Criddle, Mark S. Lacour, & Emily K. Sandoz

List of Figures & Tables

Figure S1. Participant Flow Chart by Recruitment Method. **2**
Table S1. Proportion of label type endorsement. **3**
Table S2. Regression Coefficients of AMASA on NSE Acknowledgment for Hypothesis 1........**4**
Table S3. Regression Coefficients of PF on NSE Acknowledgment for Hypothesis 2..................**5**
Table S4. Regression Coefficients of PI on NSE Acknowledgment for Hypothesis 3..................6

Figure S1. Participant Flow Chart by Recruitment Method.

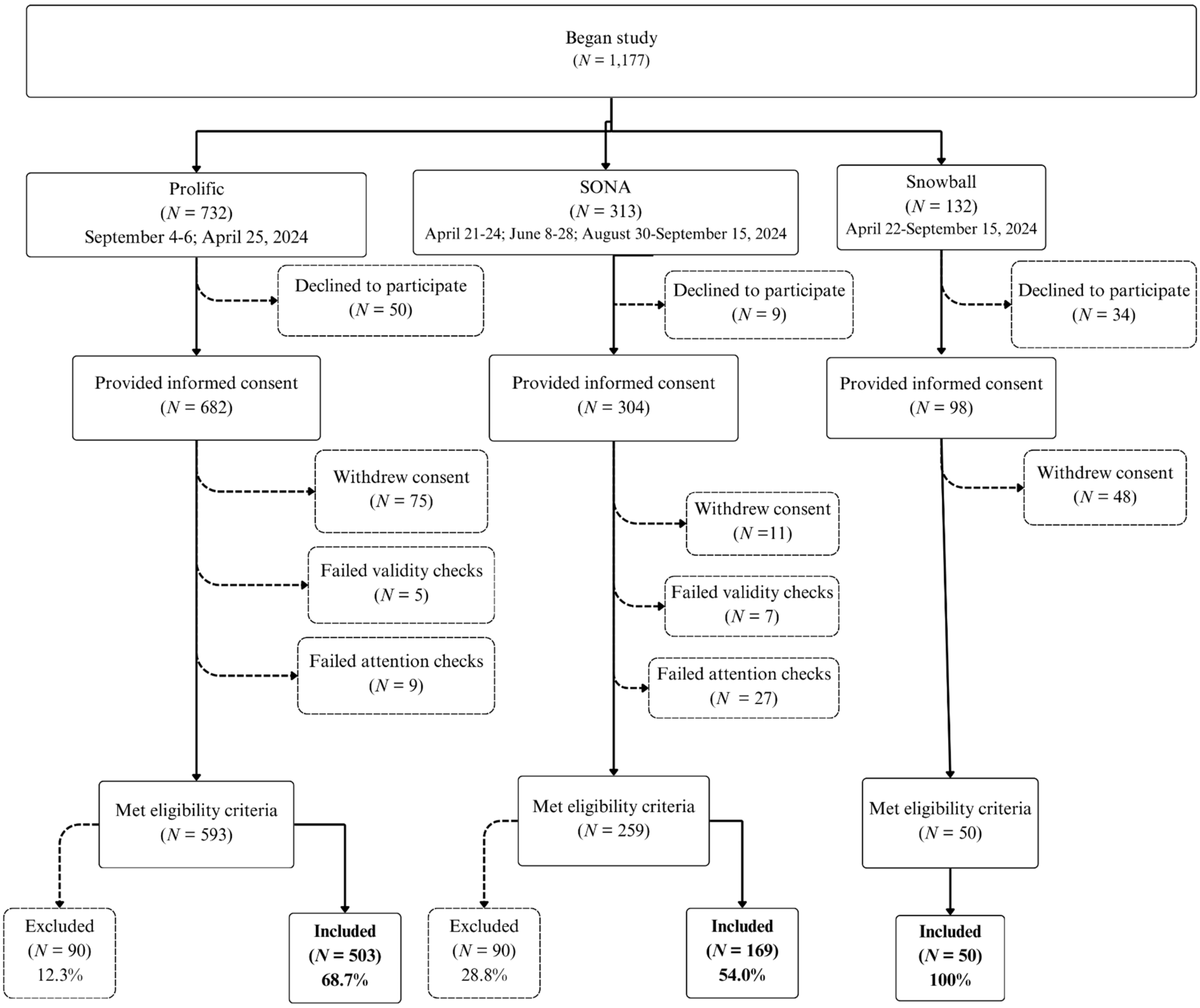

Table S1. Proportion of label type endorsement.

Label Type	Unack.		Ambiv.								Ack.	
	0		1		2		3		Total*		4	
	n	%	*n*	%	*n*	%	*n*	%	*n*	%	*n*	%
Sexual Assault [a]	177	25.4	80	11.5	69	9.9	139	19.9	288	41.3	232	33.3
Rape [b]	346	53.5	57	8.8	65	10.0	54	8.3	176	27.2	125	19.3
Acknowledgment [a]	156	22.4	75	10.8	70	10.0	136	19.5	281	40.3	260	37.3

[a] *N* = 697; [b] *N* = 647. Note. Un ack. - unacknowledged; Ambiv. = ambivalently acknowledged; Ack. = acknowledged. The discrepancies between the number of participants endorsing sexual assault and rape are due to an unforeseen technical error with Qualtrics where 49 participants did not receive the item asking if they ever experienced/perpetrated rape. * Values in this column are the sum of all the values of levels 1—3, which illustrate the total values for the ambivalently acknowledged status.

Table S2. Regression Coefficients of AMASA on NSE Acknowledgment for Hypothesis 1

Variable	*b*	*SE*	*t*	*p*	95% CI
			Model 1		
Intercept	2.38	0.06	40.39	< .001	[2.22, 2.45]
AMASA	-0.36	0.06	-6.00	< .001	[-0.47, -0.24]
R^2	0.05				
BF_{10}	> 100				
			Model 2		
Intercept	2.41	0.06	41.74	< .001	[2.24, 2.46]
AMASA	-0.52	0.06	-8.60	< .001	[-0.64, -0.40]
R^2	0.10				
BF_{10}	> 100				

Note. $N = 697$; CI = confidence interval; AMASA = acceptance of myths about sexual aggression; NSE = nonconsensual sexual experience. We examined the impact of AMASA on acknowledgment. We entered AMASA as the independent variable and acknowledgment as the dependent variable. In Model 2 ($N = 675$), we removed outliers ($n = 22$) using Cook's *D*.

Table S3. Regression Coefficients of PF on NSE Acknowledgment for Hypothesis 2

Variable	*b*	*SE*	*t*	*p*	95% CI
			Model 1		
Intercept	2.39	0.06	39.46	< .001	[2.27, 2.50]
PF	-0.05	0.06	-0.89	.37	[-0.17, 0.06]
R^2	0.001				
BF_{01}	17.70				
			Model 2		
Intercept	2.43	0.06	40.21	< .001	[2.31, 2.55]
PF	-0.06	0.07	-0.89	.37	[-0.19, 0.07]
R^2	0.001				
BF_{01}	17.43				

Note. N = 697; PF = psychological flexibility. We examined the impact of PF on acknowledgment. We entered PF as the independent variable and acknowledgment as the dependent variable. In Model 2 (N = 673), we removed outliers (n = 24) using Cook's *D*.

Table S4. Regression Coefficients of PI on NSE Acknowledgment for Hypothesis 3

Variable	*b*	*SE*	*t*	*p*	95% CI
			Model 1		
Intercept	2.38	0.06	40.11	< .001	[2.26, 2.50]
PI	0.31	0.06	5.13	< .001	[0.19, 0.42]
R^2	0.04				
BF_{10}	> 100				
			Model 2		
Intercept	2.40	0.06	40.91	< .001	[2.29, 2.52]
PI	0.41	0.06	6.76	< .001	[0.29, 0.53]
R^2	0.06				
BF_{10}	> 100				

Note. N = 697; PI = psychological inflexibility. We examined the impact of PI on acknowledgment. We entered PI as the independent variable and acknowledgment as the dependent variable. In Model 2 (N = 682), we removed outliers (n = 15) using Cook's D.
